# Spectrum Reconstruction Model Based on Multispectral Electrochromic Devices

**DOI:** 10.1002/advs.202400979

**Published:** 2024-07-12

**Authors:** Shuo Wang, Hang Yin, Yang Li, Zhen Du, Yu‐Mo Zhang, Sean Xiao‐An Zhang

**Affiliations:** ^1^ State Key Laboratory of Supramolecular Structure and Materials College of Chemistry Jilin University Changchun 130012 P. R. China; ^2^ Institute of Atomic and Molecular Physics Jilin University Changchun 130012 P. R. China

**Keywords:** electroacid/base, leuco dyes, multispectral electrochromic device, spectral camouflage, spectral reconstruction

## Abstract

Reconstructing the visible spectra of real objects is critical to the spectral camouflage from emerging spectral imaging. Electrochromic materials exhibit unique superiority for this goal due to their subtractive color‐mixing model and structural diversity. Herein, a simulation model is proposed and a method is developed to fabricate electrochromic devices for dynamically reproducing the visible spectrum of the natural leaf. Over 20 kinds of pH‐dependent leuco dyes have been synthesized/prepared through molecular engineering and offered available spectra/bands to reconstruct the spectrum of the natural leaf. More importantly, the spectral variance between the device and leaf is optimized from an initial 98.9 to an ideal 10.3 through the simulation model, which means, the similarity increased nearly nine‐fold. As a promising spectrum reconstruction approach, it will promote the development of smart photoelectric materials in adaptive camouflage, spectral display, high‐end encryption, and anti‐counterfeiting.

## Graphical abstract

With the emergence of spectral imaging, it is urgent to develop a camouflage method to confront this highly accurate detecting technology. Herein, a model and prototype electrochromic device reconstructing the visible spectra of leaves is demonstrated, which is achieved by the electroacid/base method, benefiting from the ability to regulate spectra finely and independently. This approach shows potential in spectral camouflage.

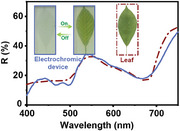

## 1 Introduction

**Figure 1:**
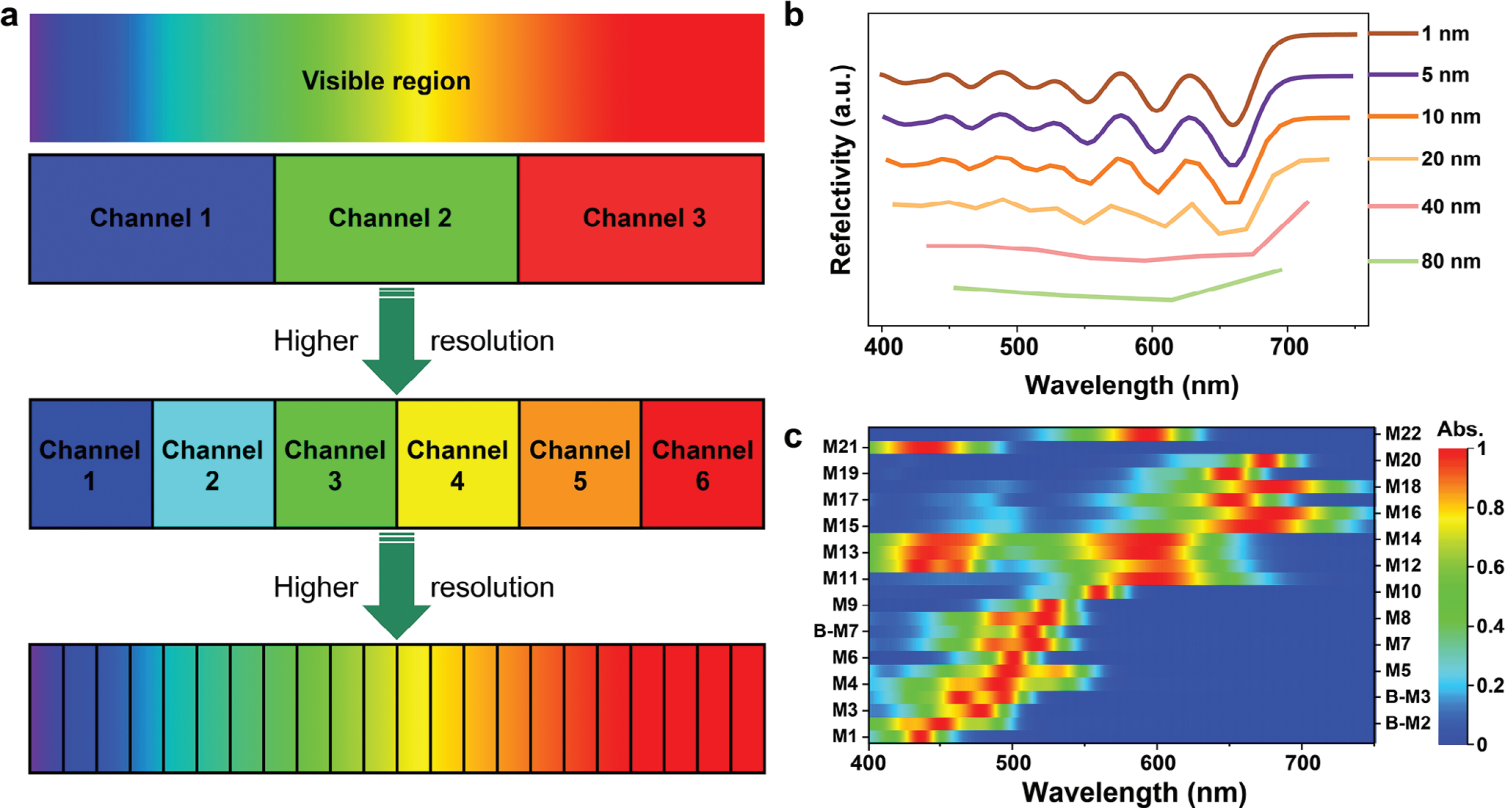
a) The schematic diagram of the multi‐channel imaging. b) The reflective spectra of object recorded every 80, 40, 20, 10, 5, and 1 nm. c) The absorption distribution of M1–M22 after treating with acid (with no prefix, like M1) or base (with the prefix “B”, like B‐M2).

In recent years, with the growing demand for high‐precision physical monitoring in both military and civilian applications, object physical detection technology and more accurate imaging technology have greatly gained development. At present, the demand is no longer satisfied with the traditional three‐channel mode of color detection and imaging, which only uses the rough spectral range of RGB (red, green, and blue). It is moving toward a narrower spectral range and more accurate with more channels mode. Such detecting and imaging technology that combines spectroscopy and imaging is called spectral imaging,^[^ ^1^ ^]^ which can scan more narrow‐band channels of objects, greatly improving the resolution of imaging (**Figure** **1**a). The more channels, the higher the spectral resolution of each sampling point, and the richer the recorded spectral information, for example, much less spectral information is recorded per 80 nm than the spectral information with higher resolution, as illustrated in Figure 1b. As a detecting technology, spectral imaging could obtain much richer information than the traditional three‐channel mode. To achieve this high‐precision scanning detection and imaging goal, multispectral (3–10 channels), hyperspectral (hundreds of channels), and ultraspectral (up to a thousand channels) imaging scanning technologies have been developed.

The rapid development of emerging spectral imaging technology has brought serious challenges to camouflage. This results in traditional color camouflage becoming no longer sufficient, as the aforementioned high‐precision spectral scanning and imaging technology can detect the spectral differences between the target and the background, which are often indistinguishable from ordinary human eyes.

Therefore, it is necessary to develop emerging spectral camouflage technology to cope and match with high‐precision spectral imaging detection technologies. Spectral camouflage is a technique that reproduces the background's spectrum near the target, in which the ability to achieve high spectral similarity to the background (like green leaves) is critical for camouflage against spectral imaging detection.^[^ ^2^ ^]^ To achieve such high‐end spectral camouflage, it is necessary to reproduce the target's spectrum as closely as possible with the actual background. If the target's spectrum is similar enough to the background in each channel (band) interval, the imaging technique is not able to distinguish the target from the background, so spectral camouflage is achieved. The higher the level of spectral imaging and detecting, the higher the matching requirement for the target spectrum in each smaller band interval, which requires the target's spectrum highly adjustable.

Among numerous candidates for the above goal, electrochromic materials have the potential to meet these needs. Electrochromic materials undergo redox under electrical stimulation, causing the color (spectrum) change. The current electrochromic materials and devices exhibit enormous potential for spectral camouflage due to their structural diversity^[^ ^3^, ^4^, ^5^, ^6^, ^7^, ^8^, ^9^, ^10^, ^11^ ^]^ and color diversity,^[^ ^12^, ^13^, ^14^, ^15^ ^]^ so they could be used to reconstruct the spectrum of devices to reproduce the desired spectrum of targets. The development of multispectral electrochromic materials also has great application potential in adaptive camouflage,^[^ ^16^, ^17^, ^18^, ^19^ ^]^ advanced anti‐counterfeiting,^[^ ^20^, ^21^ ^]^ and so on. Some outstanding researchers developed electrochromic materials and devices with rich color (spectrum), like a painter's palette.^[^ ^22^, ^23^, ^24^, ^25^ ^]^ However, electrochromic materials still need to be improved in some aspects to achieve a better spectral camouflage effect. First, the number of spectra achieved by one electrochromic material system needs to be enriched, which can increase the number of bands that can be utilized and regulated. The second is that the current electrochromic material's FWHM (full width at half maxima) is usually wide,^[^ ^8^, ^12^, ^24^ ^]^ which is not conducive to the fine regulation of the bands. The third is the ability to regulate the absorption peak independently and precisely. The redox of current electrochromic materials is usually accompanied by a multi‐change of the absorption band,^[^ ^5^, ^10^, ^26^ ^]^ which is not helpful to the independent tuning of the bands. Those are related to the inherent mechanism of electrochromic materials, so it is difficult for them to be applied in the field of spectral camouflage.

In this paper, we developed a spectral simulation model and simulated the visible spectrum of a real object using a newly developed electrochromic system based on the PCET (proton‐coupled electron transfer) mechanism, called the electroacid/base method.^[^ ^27^, ^28^, ^29^, ^30^ ^]^ This system is based on intermolecular PCET, using p‐phenylenediamine derivatives as the electroacid, BQ (p‐benzoquinone) as the electrobase, and acido/basochromic dyes as the color‐changing unit, where the electro‐active unit and color‐changing unit are separate, and thanks to that, the spectrum of this system is highly adjustable. Over 20 spectra (bands) were achieved by different leuco dyes (Figure 1c, dye molecular engineering details in Supplementary Note 1: molecular design, Figure S1 and Supplementary synthesis methods), their absorption uniformly distributed within the visible region, each of which can be independently regulated for multispectral regulation. The simulation process was shown and the variance had gone down ≈90%.

## 2 Experimental Section

**Figure 2:**
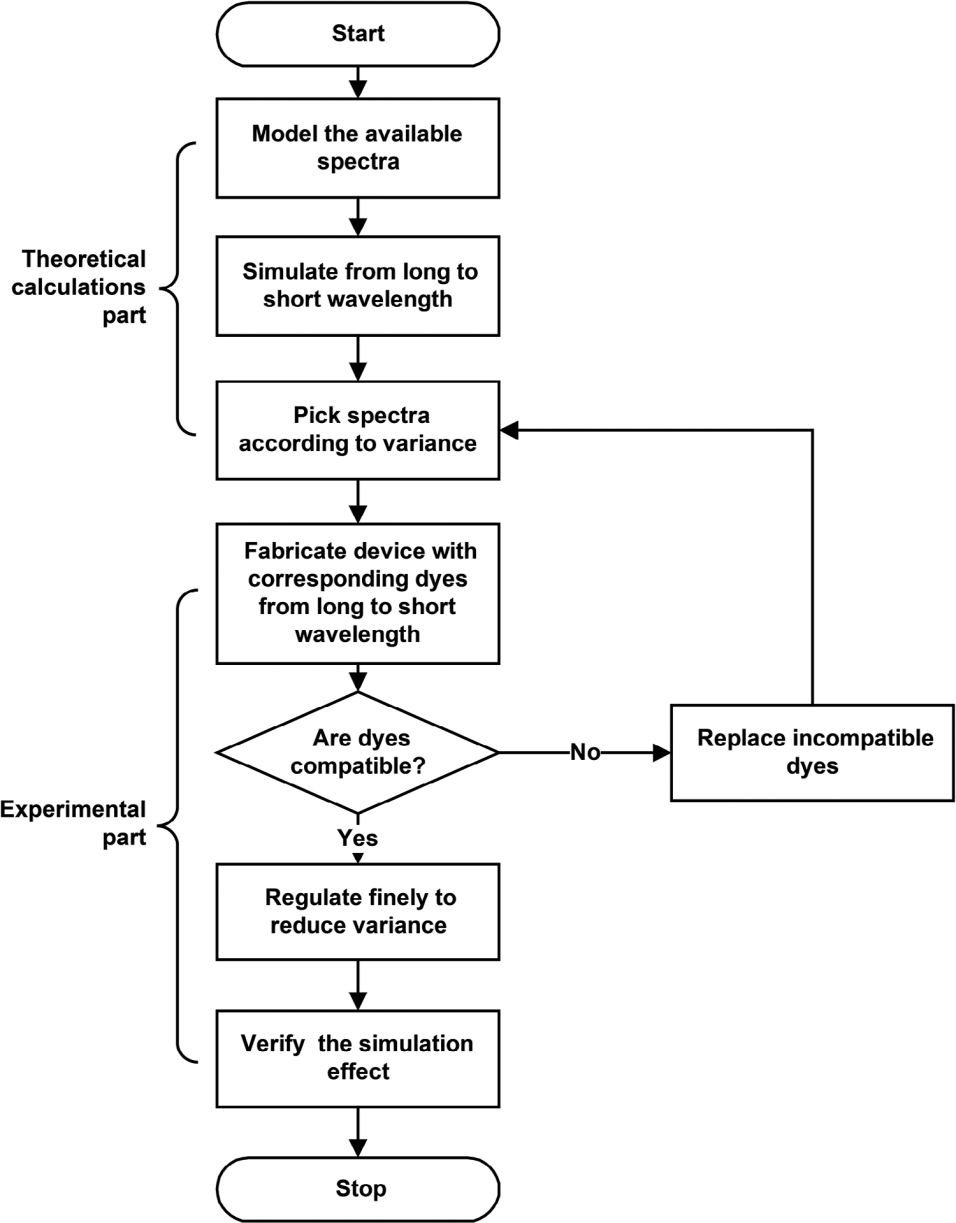
The flow chart of the simulation model.

The simulation model was used to calculate and determine the spectra of one system used to simulate real objects. The whole process was divided into two parts in **Figure** **2**. First, the theoretical optimal spectra combination to simulate real objects are obtained by mathematical calculations called the “theoretical calculations part”. And then the spectra combinations are verified by experiments, such as optimizing the concentration ratios of the dyes, which was called the “experimental part”. Here, in order to quantitatively analyze the difference, the population variance “*σ^2^*” between those two spectra was introduced, and was calculated by the equation:

(1) $$\sigma^{2}=\frac{\sum {\left(X-\mu \right)}^{2}}{N}$$

where *σ^2^* was the population variance, *X* was the difference of reflectance or transmittance (%) under the same wavelength, *µ* was the popular mean of the differences and *N* was the total number of the differences. The variance could measure the dispersion of a set of numbers, and how far the set of numbers was spread out from their average value. Variance analysis could determine the matching degree of two spectrum curves, that is, the better the spectrum of the device and object matches, the smaller the variance was, which means a better simulation effect.

### 2.1 The Theoretical Calculations Part

First, all available spectra are modeled to obtain the functions f(x) of their spectrum (absorption vs wavelength), which will be used to simulate the spectra of real objects. Here the spectra of 22 leuco dyes after treatment of acid or base were modeled to the mathematical formula f(x), shown in Figure 1c, Figure S1 (Supporting Information) and Supplementary functions. Then, the simulation was started from a long wavelength to a short wavelength. The weights [a] as variables were assigned to f(x) and the reflective spectra were replaced with 10^−af(x)^. Calculate the variance between the spectrum of a real object and the simulated one, select the simulation spectrum with the smallest difference for the long wavelength region, and choose its corresponding dye here. Then the simulation moved forward to the following experimental part. After the determination in the experimental part, the theoretical calculation moved forward to the short wavelength.

### 2.2 The Experimental Part

After the theoretical calculation, the experimental part should be carried out to determine the feasibility of the electrochromic system. Here, the electrochromic system based on the PCET mechanism was selected to perform this part, because of its rich spectral selectivity as mentioned previously.

In this part, the electrochromic device was fabricated using the corresponding dyes screened out in the theoretical calculations part, in which the dye species were added gradually from long to short wavelengths, to determine whether they were compatible. If the dyes were not compatible, the experiment will return to the theoretical simulation part, those incompatible dyes will be replaced by the next fitting one according to the theoretical calculations part, the experiment was performed again till the simulation effect got the best. The variance was continuously reduced so that the spectra of the device and object was increasingly matched. There were some parameters that may cause the incompatible problem, such as the working potential, molar absorptive coefficient, and so on. Thus, the experimental part was needed to determine whether the combinations of spectrum feasible in the device.

In the end, the fine regulation was executed to minimize the variance using the combination of spectra obtained above. After the simulation effect was verified, the simulation process was finished.

### 2.3 The Simulation of Real Objects

Here, the spectral simulation of leaves was demonstrated and discussed the process of reconstructing the spectra of them. Vegetation is the most typical and common environmental ground feature, and the reconstruction of its spectrum is valuable for the spectral camouflage of ground targets. Leaves are common natural objects of vegetation in the daily lives, active and static pigments, microcapsule colorants and generic bilayer coating methods have been developed by excellent researchers to realize the spectral camouflage of natural leaves.^[^ ^2^, ^31^ ^]^ Rather than static color and fixed spectrum, electrochromic materials could offer the color change under bias and switch between desired spectra like “off” or “on” camouflage states flexibly. Though the L*a*b* values of leaves have been reappeared by some outstanding researchers^[^ ^18^, ^19^ ^]^ using electrochromic materials, the visible spectra of leaves are reproduced with great challenge with electrochromic materials.

**Figure 3:**
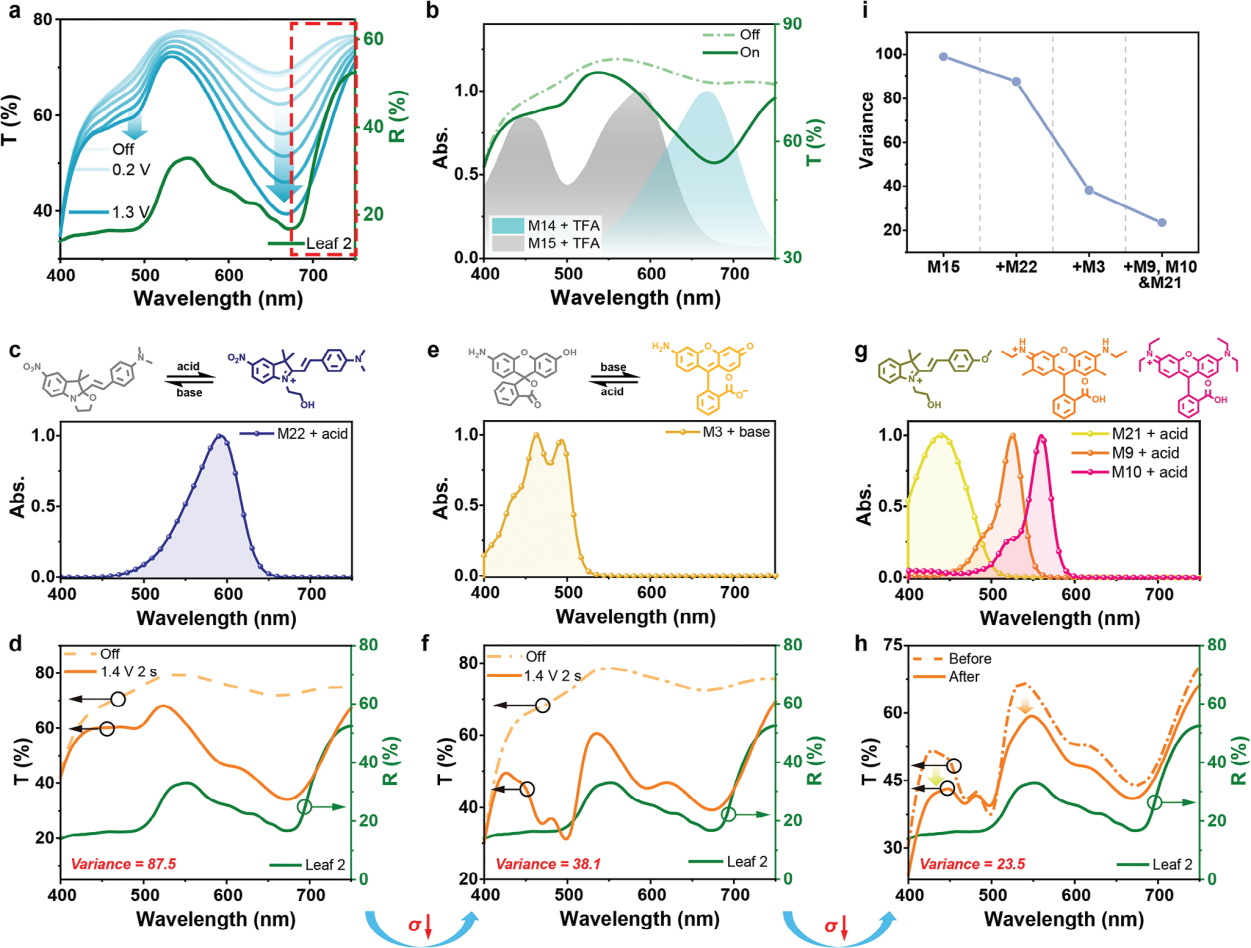
a) The visible reflective spectrum of leaf 2 (green line) and the transmittance spectra of device containing M15 and DMPA (cyan line, treated with +0.2–+1.3 V for 1 s). b) The transmittance spectra of device containing “DMPA + M14 + M15” (+1.4 V 2.0 s, electrochromic layer solution: M15: 2*10^−3^, M14 and DMPA: 1*10^−2^ M) and the absorption spectra of M14 (grey shadow) and M15 (cyan shadow) treated with TFA (trifluoroacetic acid). c) The absorption spectra of M22 treated with TFA and the corresponding structural change. d) The transmittance spectra of device made with “DMPA + M15 + M22” (electrochromic layer solution: M15: 4*10^−3^, M22: 2*10^−3^, DMPA: 1*10^−2^ M) and the reflective spectrum of leaf 2 (green line). e) The absorption spectra of M3 treated with TBAOH (tetraethyl ammonium hydroxide) and the corresponding structural change. f) The transmittance spectra of device using M3 in ion storage layer and reflective spectrum of leaf 2 (green line). g) The absorption spectra of M21, M9, and M10 treated with TFA and the corresponding structure respectively. h) The transmittance spectra of device before and after introducing M9, M10, and M21 in electrochromic layer and fine adjustment of concentration and reflective spectrum of leaf 2 (green line). i) The change of experimental variance during the spectrum reconstruction process.

To determine the reflective features of leaves, several common leaves were collected and their visible reflective spectra were measured (Figures S2 and S3, Supporting Information), they exhibited similar features. Taking leaf 2 (elm leaf) as an example, the key to reconstructing its spectrum is to simulate the following two features: the red edge at 680–750 nm^[^ ^32^ ^]^ (**Figure** **3**a green line in red box area), and a green reflective feature at ≈550 nm (Figure 3a green line).

Taking the simulation of leaf 2′s spectrum as example, first all the spectra of dyes were modeled to give their f(x) (Figure S1, Supporting Information and Supplementary functions). The simulation process started from the long‐wavelength (550 nm–750 nm) and the variance between spectrum of dyes and leaf 2 was calculated using theoretical methods. M15 was chosen to simulate the red edge area due to its smallest theoretical variance (50.7 at 550 nm–750 nm). The authors think its proper λ_max_ (maximum absorption wavelength) at 668 nm and FWHM (112.7 nm) makes it match the feature of red edge well. The device containing M15 and DMPA (methyl 2‐((4‐(dimethylamino)phenyl)amino)benzoate) as electroacid^[^ ^28^, ^29^ ^]^ was fabricated (details in Methods, device structure in Figure S4 (Supporting Information) and Synthesis methods in SI), DMPA could release/capture proton under bias to switch the leuco dye between bleached and colored states, the absorption of these leuco dyes results from ICT (intramolecular charge transfer), treated with acid/base, these dyes form large conjugated system with delocalized electrons and electron‐donor/acceptor group, which leads to strong ICT absorption, and their absorption could be finely tuned by molecular design (details in Supplementary Note 1: Molecular design) to cover the visible region. The electrochromic mechanism of DMPA as electroacid is shown in Figures S5 and S6 (Supporting Information). The optical modulation could be realized finely through the regulation of concentration and bias applied (Figure 3a; Figure S7, Supporting Information). The experimental variance between the spectra of the device (Figure 3a cyan line) and leaf 2 (Figure 3a green line) was 98.9. Such a big variance was due to the mismatch of the spectra in 550 nm–670 nm and 400 nm–550 nm.

M15 could not offer enough absorption at 550 nm–670 nm, and another dye was needed to assist M15 realizing better simulation. Through the theoretical calculation part, M14 was chosen to realize better simulation effect with M15 (theoretical variance: 11.1 at 550 nm–750 nm). However, proceeding to the experimental part, M14 exhibited no electrochromic property when mixed with M15 in the device (Figure 3b green line). This phenomenon also occurred when M11 or M12 mixed with M15 (Figure S8, Supporting Information), which may be due to the big difference of the sensitivity to proton (details in Figures S9 and S10), M15 is much more sensitive to proton than M14 so that they were not compatible in the electrochromic layer. Therefore, we returned to the calculation part, and M22 was chosen (Figure 3c). The combination of M22 and M15 had the theoretical variance of 12.5 at 550 nm–750 nm. Though it's larger than the combination of M14 and M15 theoretically, M22 and M15 were compatible working together experimentally. And then in the experimental part, the device containing M15 and M22 showed good simulation effect at the long‐wavelength region, along with a favorable decrease in experimental variance (from 98.9 to 87.5) (Figure 3d). Thus, the combination of M14 and M15 was selected to simulate the feature at long‐wavelength (550 nm–750 nm) region.

At the short‐wavelength region, M3 was selected to simulate the spectrum from 400 to 550 nm, which exhibits both acidochromic and basochromic (Figure S11). The absorption spectrum of M3 after basochromic process is broad (FWHM = 76.4 nm) to cover this region (Figure 3e) with the theoretical variance (104.4 at 400 nm–550 nm). The addition of M3 results in better spectral matching between the device and leaf 2, and the experimental variance between them further reduced (from 87.5 to 38.1) (Figure 3f). It should be clarified that electrochromism of M3 was achieved by mixing with BQ as electrobase^[^ ^30^ ^]^ in ion storage layer, thus double‐layer electrochromic device was achieved (device structure in Figure S12). BQ as electrobase could achieve electrochromism with basochromic leuco dyes and the optical modulation also could be realized finely through the regulation of concentration and bias applied in device (Figures S13–S15).

### 2.4 Fine Regulation and Prototype Device

After the major decrease of the experimental variance, the concentration of dyes was adjusted finely to regulate the relative intensity at different wavelength, bigger concentration of dye led to stronger absorption intensity (lower transmittance/ lower reflectivity) at its wavelength (details in Figure S16). And several dyes (Figure 3g) were added in small amount at the electrochromic layer to finely regulate and smooth the spectrum (Figure 3h), resulting in the experimental variance decreased from 38.1 to 23.5 (then decreased to 10.3 in the following reflectance test). Thanks to the rich choice of dyes with different absorption spectrum and the ability to regulate spectra independently, the experimental variance of the spectra between device's and leaf's decreased gradually (Figure 3i). Some dyes used here are strongly fluorescent in solution, but the influence of fluorescence of them on device was negligible (details in Figure S17).

**Figure 4:**
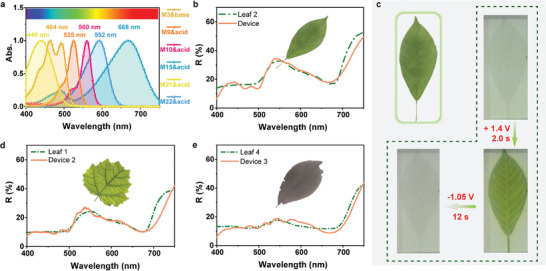
a) The absorption spectra of the dyes introduced for simulation of leaf. b) The visible reflective spectra of the device treated with +1.4 V for 2.0 s (orange line) and leaf 2 (green dashed line) (inset: the photo of leaf 2). c) The photos of leaf 2 (top‐left) and the device before and after electrochromism. d) The visible reflective spectra of device 2 treated with +1.6 V for 4.0 s (orange line) and leaf 1 (green dashed line) (inset: the photo of leaf 1). e) The visible reflective spectra of device 3 treated with +1.5 V for 3.0 s (orange line) and leaf 4 (green dashed line) (inset: the photo of leaf 4).

Finally, six kinds of dyes with different characteristic absorption were employed to reconstruct the visible reflective spectra of leaf 2 (**Figure** **4**a). Here, DMPA as electroacid could work with M9, M10, M15, M21, and M22 to achieve electrochromism in electrochromic layer, and BQ as electrobase could work with M3 to achieve electrochromism in ion storage layer at the same time. In order to verify the effect of spectral reconstruction, the electrochromic device with the optimal combination and concentration of dyes was fabricated with the laser etched ITO (indium tin oxide) glass with the pattern of leaf 2 (Figure S18). As shown in Figure 4b, the device exhibited similar visible reflective spectrum with leaf 2′s after +1.4 V for 2.0 s (experimental variance: 10.3), and its green color and pattern were also vivid (Figure 4c). After the stimulation of reverse voltage, the device recovered to its initial nearly colorless state (Figure 4c). The device performed coloring‐bleaching cycles over 780 times with 4.4% attenuation in ΔT (transmittance change) (Figure S19). Weather resistance test showed the device maintained the ability to simulate leaf 2 under +50 or −10 °C for 3 h (Figure S20). Leaves have similar spectral features, their spectra may be simulated by the same combination of dyes in Figure 4a in this method. Furthermore, through the adjustment of concentration of dyes shown in Figure 4a and the electrical stimulation (details in Methods), the spectral reconstruction of the yellowish green leaf 1 (Figure 4d, experimental variance: 13.1) and dark red leaf 4 (Figure 4e, experimental variance: 8.8) were achieved through another two electrochromic devices after bias applied.

Therefore, the reconstruction of leaf's reflective spectra was realized through the spectra (dye)‐mixing way in electrochromic device.

## 3 Methods

### 3.1 Materials

Unless otherwise stated, all the chemicals and reagents were purchased from Energy Chemical and used as received without further purification. Acetonitrile (MeCN) and tetrahydrofuran (THF) were chromatographic grade. Tetrabutylammonium hexafluorophosphate (TBAPF_6_) was recrystallized from ethanol three times and dried under vacuum. Dye molecules and DMPA were synthesized as described in Supporting Information.

### 3.2 Preparation of Device

The solution of three layers was prepared as following:

### 3.3 Electrochromic Layer

Polymethyl methacrylate (PMMA, 180 mg), propylene carbonate (PC, 37.5 µL), TBAPF_6_ (75 mg) and THF (1.0 mL) were mixed together and stirred till PMMA was completely dissolved. Then DMPA and dyes were added accordingly.

### 3.4 Ion Conducting Layer

PMMA (10.8 g), PC (2.25 mL), TBAPF_6_ (4.5 g), and MeCN (60 mL) were mixed together and stirred till PMMA was dissolved.

### 3.5 Ion Storage Layer

PMMA (0.9 g), PC (0.1875 mL), TBAPF_6_ (0.375 g), and MeCN (10 mL) were mixed together and stirred till PMMA was dissolved. Then 2,2,6,6‐tetramethylpiperidinooxy (TEMPO, 156 mg) and BQ (54 mg) were dissolved in the solution. Then dyes were added accordingly.

The electrochromic layer solution used to simulate leaf 2 was then added M15 2.03 mg, M22 0.76 mg, M9 0.21 mg, M10 0.22 mg, M21 3.21 mg, DMPA 2.70 mg; The electrochromic layer solution used to simulate leaf 1 was then added M15 2.03 mg, M22 0.8 mg, M10 0.22 mg, M9 0.3 mg, M21 2.8 mg, DMPA 2.70 mg; The electrochromic layer solution used to simulate leaf 4 was then added M15 3.04 mg, M22 2.08 mg, M10 0.53 mg, M9 0.75 mg, M21 2.25 mg, DMPA 2.70 mg.

The ion storage layer solution used to simulate leaf 2 was then added M3 33.1 mg; The ion storage layer solution used to simulate leaf 1 was then added M3 5.00 mg; The ion storage layer solution used to simulate leaf 4 was then added M3 5.00 mg.

## 4 Conclusion 

The simulation model to mimic the spectra of real objects was proposed, and the process of reconstructing visible spectra of leaves was demonstrated by the electrochromic system based on electroacid/base method. Over 20 kinds of pH‐dependent leuco dyes have been synthesized/prepared through molecular engineering to provide available spectrum/band to optimize the similarity between the spectra of device and leaf. Fortunately, the spectral variance between the device and elm leaf was reduced from 98.9 down to 10.3 by adjusting precisely and finely the spectra of electrochromic devices containing leuco dyes, electroacid and electrobase. The spectrum reconstruction approach based on proposed simulation model and electrochromic materials may accelerate the development of adaptive spectral camouflage.

## 5 Discussion

In the theoretical part, the optimal combination of spectra to simulate real objects could be calculated, but there may be incompatibilities among the corresponding materials. In this study, we determined that there was difference in the degree of dyes responding to proton so that dyes may be incompatible in one layer, which may be addressed in further work by the development of electroacid or electro‐lewis‐acid with smaller pKa. Then, in order to make this method versatile and flexible, we propose a general model, which is more efficient to simulate objects by mathematical calculations to seek appropriate match of dyes, and gives the most suitable dyes match to simulate real objects from the viewpoint of mathematics (see Supplementary method 4: The universal model).

Besides, this study only talked about the spectral simulation in the visible region, the simulation of NIR (near infra‐red) region need to be addressed in further work.

As the materials with highly adjustable spectra, we demonstrated its feasibility in spectral camouflage by reproducing the spectra of objects, and here are some other ideas for its further study and potential application:

1. Spectral displays. Compared with RGB‐based display using only three spectral bands, multi‐spectral displays can reconstruct the visible spectrum and reproduce the color as accurately as possible by minimizing the spectral difference between the displayed and the desired spectrum while maximizing the visual quality.
2. High‐end anti‐counterfeiting. The spectrum of the device based on this system is highly adjustable, depending on the dyes used. The goods is real only if its spectra matches the standard spectrum.
3. High‐end encryption and information storage or delivery. The system has many channels in the visible region, each channel could represent a symble (information) like 1, 2, 3, 4 and so on, and the absorption (transmittance or reflectance) intensity could be devided into several interval, represented by a, b, c, d and so on. Then the spectrum of the device based on this system could carry (store) and express abundant information using the combination provided by the dyes.

The electrochromic performance should be improved to be applied in the field mentioned above, for example, long term bistability is needed for anti‐counterfeiting and information storage, and fast response is needed for the high refresh rate scene like spectral display and information delivery.

## Conflict of Interest

The authors declare no conflict of interest.

## Author Contributions

Y.‐M.Z., S.X.‐A.Z., and S.W. conceived this project. S.W. and Y.‐M.Z. designed the experiments. S.W., Y.L., and Z.D. synthesized the molecules. S.W. performed the experiments and researched the mechanism. H.Y. built the instrument platform for the measurement of reflectivity. S.W., Y.‐M.Z., and S.X.‐A.Z. analyzed the results. S.W., Y.‐M.Z., and S.X.‐A.Z. wrote and revised the manuscript. The project was planned, directed and supervised by S.X.‐A.Z., and Y.‐M.Z. All authors have given approval to the final version of the manuscript.

## Supporting information

Supporting Information

## Data Availability

The data that support the findings of this study are available from the corresponding author upon reasonable request.
