# Supplementary material for: Spectrum Reconstruction Model Based on Multispectral Electrochromic Devices
Source: Adv Sci (Weinh). 2024 Jul 12;11(35):2400979. doi: 10.1002/advs.202400979 (PMC11425250; doi:10.1002/advs.202400979)
Supplement: Supplementary file 1 — Supporting Information [file ADVS-11-2400979-s001.pdf]

Supporting information

Spectrum reconstruction model based on multispectral electrochromic devices

Shuo Wang, Hang Yin, Yang Li, Zhen Du, Yu-Mo Zhang, and Sean Xiao-An Zhang**

S. Wang, Y. Li, Z. Du, Y.-M. Zhang and S. X.-A. Zhang

State Key Laboratory of Supramolecular Structure and Materials, College of Chemistry, Jilin University, Changchun, 130012, P. R. China.

E-mail: zhangyumo@jlu.edu.cn and seanzhang@jlu.edu.cn

H. Yin

Institute of Atomic and Molecular Physics, Jilin University, Changchun, 130012, P. R. China.

Contents

Supplementary methods: 3

Supplementary notes: 5

Note 1: Molecular design 5

Supplementary figures 9

Supplementary functions 23

Synthesis methods 31

Supplementary reference 53

Supplementary methods:

Supplementary method 1: Instrument information

^1H NMR and ^{13}C NMR spectra were recorded with Wuhan Zhongke Niujin As 400 and Bruker AVANCE500 at room temperature, the data was reported in ppm relative to tetramethylsilane (TMS) as internal standard; The LC-HRMS data was obtained from Agilent 1290-microTOF-Q II mass spectrometer using sodium formate as internal standard; The Maldi-Tof-MS data was obtained from Brucker Autoflex speed TOF mass spectrometer; The absorption/transmittance spectra were recorded on Shimadzu UV-2550 double-beam spectrophotometer; The electrochemical data was recorded on Bio-Logic： SP-150; The ion storage layer and ion conducting layer were blade coated on ITO electrode by automatic film application BEVS1811; The reflective spectra were recorded by Maya 2000 Pro spectrometer and a DH-2000-BAL balanced, deuterium and tungsten halogen light source, calibrated with STD-WS.

Supplementary method 2: Cyclic voltammetry (CV) experiments

CV experiments were performed under room temperature (20 ^oC) using three-electrode system. The working electrode was glass-carbon electrode (3 mm dia.), polished with alumina (0.3 μm and 0.05 μm). The counter electrode was platinum wire, and the reference electrode was Ag wire. The solvent was chromatographic grade acetonitrile and 0.1 M $TBAPF_6$ was added as supporting electrolyte, the tested molecules were dissolved in the solution and the concentration was 1.0 * 10^{-3} M. The scan rate was 100 mV/s. The CV was calibrated by ferrocene.

Supplementary method 3: In-situ spectroelectrochemistry test

The light path of the cell was 1 mm, the working electrode was platinum net placed in the light path of the cell. The counter electrode was platinum wire, the reference electrode was Ag wire. The solvent was chromatographic grade acetonitrile and 0.1 M $TBAPF_6$ was dissolved as supporting electrolyte.

Supplementary method 4: The universal model

1. Model for every spectrum from Fig.S1 mathematically to get the f (function) of λ (wavelength, nm), e.g. $A_{M1} = f_{M1}(\lambda)$, where A_{M1} is the absorption of M1, all the models were given as functions at Supplementary functions.
2. Give weight ($a_1, a_2, a_3, \dots, a_{22}$) to every function, add the functions at the same wavelength to the absorption

at 400 nm : $A(400) = a_1f_{M1}(400) + a_2f_{M2}(400) + a_3f_{M3}(400) + \dots + a_{22}f_{M22}(400)$

at 401 nm : $A(401) = a_1f_{M1}(401) + a_2f_{M2}(401) + a_3f_{M3}(401) + \dots + a_{22}f_{M22}(401)$

…

at 750 nm: $A(750) = a_1f_{M1}(750) + a_2f_{M2}(750) + a_3f_{M3}(750) + \dots + a_{22}f_{M22}(750)$

3. Transfer A{400, 401, … , 750} (the absorption at every wavelength) to T{400, 401, … , 750} (transmittance at every wavelength) according to the Lambert-Beer's law

$A = -lgT, T = 10^{-A}$.

4. Calculate the variance between the above-mentioned T{400, 401, … , 750} and the reflectance of the to-be simulated object.

5. Take the set of weight (a_1, a_2, a_3, …, a_{22}) which gives the smallest variance.
6. Prepare the electrochromic layer solution according to the weight (a_1, a_2, a_3, …, a_{22}) and fabricate the device, adjust the molar ratio of the dyes accordingly.

Supplementary notes:

Note 1: Molecular design

In this work, leuco dyes are chosen as coloring-unit because: 1. Dyes usually have high molar absorption coefficient among compounds with color, and that leads to high coloration efficiency in electrochromic system, which makes the electrochromic device coloring more efficiently and saves more energy. 2. Leuco dyes are colorless initially, and will color under stimulation like acid/base, that provides us the device with off (colorless) and on (colored) states, which offers the device more possibilities like military deception. 3. Leuco dyes with acido/basochromic property were selected in this paper to work with electroacid/base to achieve electrochromism, causing our device could switch between colorless and colored state under bias with desired spectra like “off” or “on” camouflage states flexibly, their spectrum is not fixed like normal compounds with color.

Xanthene molecular switches can be modulated between colored and bleached state through controlling the switch of the lactone part, the color/spectra modulation is usually performed on the xanthene part. Traditional xanthene dyes can’t cover the full visible spectrum after the acidochromic process. Rh-B (Rhodamine B, M10) shows the λ_{max} (maximum absorption wavelength) at 550 nm. Conventional modifications of rhodamine are often performed on the 3' and 6' positions (Fig. S1b R_1 and R_2), which can strongly change the color of such molecules, for example, performing a modification of methoxy group at both positions can yield yellow xanthene molecules (M1, λ_{max} = 435 nm). Following, enhancing the electron-pushing ability of these two position’s groups or lengthening the conjugated structure often results in molecules with red-shifted absorption. For example, ethylamino group can redshift the λ_{max} to 525 nm (Rhodamine 6G, M9, FWHM = 31.8 nm), diethylamino modification can redshift it to 550 nm (Rhodamine B, M10, FWHM = 32.1 nm), and di-p-tolylamino group modification can redshift it to 595 nm (M11, FWHM = 99.5 nm). Traditional rhodamine-based xanthene molecules can cover blue to yellow absorption region, but blue molecule (M11) obtained by the strategy of lengthening the conjugate length has relatively wider FWHM. This strategy introduces redshift of dyes, while also introduces more orbitals, broadening the absorption peak of the molecule.

Recent times, some researchers have jumped out of the shackles of traditional xanthene leuco dyes

structure and extended the spectral range of such pH indicators (Fig. S1c, 1d). For example, some researchers gave up part of the rigidity of the molecular skeleton and increased the conjugate length further, which resulted in the molecule's absorption red-shifted to 668 nm (M15). Furthermore, if the skeleton rigidity on one side is further broke, the λ_{max} can be further red-shifted to 682 nm (M18). The λ_{max} of these molecules can be more finely tuned by modifying the length of the alkyl chain of the amino substituent at both ends of the molecule or by cyclizing it. In addition, in order to obtain other properties, researchers have made more modifications on this structure, such as modifying coumarin on such molecules to obtain the switch in fluorescence properties, while obtaining a λ_{max} of 651 nm (M17).

Above all, by modifying the push/pull electron strength, the absorption can be red-shifted to ~550 nm at most. All subsequent redshifts are resulted from lengthening the conjugate length of the molecule or weakening the rigidity of the molecular structure, while the absorption peaks obtained by this strategy are broadened at the same time. In addition to changing the push/pull strength, changing the conjugate length, and changing the molecular rigidity, changes can also be made to the heteroatom oxygen in the main coloring body of xanthene. The oxygen atom here ensures the rigidity of such molecules (Fig. S1b), and if the oxygen atom is not present, then the absorption of the molecule is further red-shifted, as in the comparison of malachite green lactone and Rhodamine B, for example. Furthermore, replacing the oxygen atom with other atom will generally cause the absorption to red-shift. For example, replacing the oxygen atom with a dimethyl-substituted methylene (λ_{max} = 612 nm), or with a dimethyl-substituted silicon (M19, λ_{max} = 649 nm, M20, λ_{max} = 674 nm), or phosphorus (λ_{max} = 695 nm *ca.*), or sulfur (λ_{max} = 703 nm), can greatly redshift the absorption of the molecule to the red region. Moreover, this approach of replacing the main body heteroatom does not lengthen the conjugate structure of the molecule, so the obtained absorption is red-shifted, while with a small FWHM. The longer conjugate length can be considered if a wider spectral coverage is desired. The same can be done on the substituents at the ends of the molecule to finely tune the λ_{max}, for example, by adjusting the alkyl chain length or by cyclizing it (M20).

Particularly, fluoran leuco dyes can achieve a wide spectral coverage. Fluoran dyes can achieve double absorption peaks in the visible region and thus overlay to achieve colors such as black, green, etc. These dyes often have substitutions at the 3' (R_1), 6' (R_2), and 7' (R_4) positions, such as ODB-2 (M14), TFG (M13), and CK-5 (M12). The double absorption peaks at ~450 nm and ~590 nm can

provide a wide spectral coverage if needed.

For basochromic switches, it is necessary to introduce fluorescein-based dyes. Fluorescein loses a proton when it encounters base, and when the negative charge resonates to the quaternary carbon, the oxygen linkage between the quaternary carbon and the lactone breaks to form a more stable ring-opening structure with carboxylate, at which point the conjugation at both sides of the xanthene is connected, which brings strong color change. And if the alkali strength is further enhanced and the molecule continues to lose a proton, then the absorption of the molecule is strongly enhanced and red-shifted. During the process, the λ_{max} will gradually red-shift from ~450 nm to ~500 nm. The common tetrachloro, tetrabromo and tetraiodo substituted fluorescein can broaden the absorption spectrum of fluorescein and introduce a change at λ_{max} at the same time. In addition, modifications on the bottom ring (lactone located benzene ring) can also change the λ_{max} of the molecule and the ease of switching the ring open and close. Fluorescein molecule loses protons in two steps, the λ_{max} will be changed during the regulation process, if only the absorption intensity change is desired, while the λ_{max} keeps constant as the amount of alkali increases, the phenolic hydroxyl group on one side should be methylated (Fig. S1b, M2), so that the λ_{max} of the molecule is fixed at 450 nm (M2). The absorption peak position can be red-shifted by replacing the phenolic hydroxyl group on one side with an amino group (M3, λ_{max} = 464 nm for basochromic, 478 nm for acidochromic), diethylamino group (M7, λ_{max} = 512 nm for basochromic, 517 nm for acidochromic), etc.

There are many other basochromic switches. Phenolphthalein and sulfophthalide dyes are basochromic, and similarly as above, their λ_{max} can be adjusted by changing the substituents on both end groups of the molecule and by modifying the benzo ring. However, the molecule of sulfophthalides seems difficult to be stabilized at their colorless state, and such molecules are easily stabilized in the state of zwitterion due to the presence of sulfolactone rings, where such molecules are generally yellow, further loss of protons introduces red shift, and shows mixed color after overlay with yellow (e.g. bromocresol green is green at this time), and with further proton loss, the yellow state disappears and shows red-shifted color (e.g. bromocresol green is blue at this time). Since these molecules are not colorless in the pristine state, they do not belong to leuco dyes in a sense and are not discussed further. In addition, some small molecules with phenolic hydroxyl groups can also be basochromic, such as nitro phenols, hydroxycoumarins, hydroxyquinolines, etc., but the molar absorbance coefficients of these small molecules are generally small, so they are not

discussed further in this article as well.

The molecules above can also be modified on the benzene ring to adjust the absorption wavelength, peak shape, ring opening ability, etc. For example, halogen modification on the chromogenic ring can broaden the absorption peak, and nitro modification on the bottom ring can adjust the ease of ring opening.

Furthermore, oxazoline-based switches are also excellent leuco dyes (Fig. S1f). These molecules also have the advantages of high molar absorbance coefficients and easy modification to achieve multispectral modulation. The coloring and bleaching of these molecules are achieved by the opening or closing of the oxazoline ring. The opening of the oxazoline ring connects the conjugation on both sides of the oxazoline ring and brings strong absorption change. The λ_{max}/spectrum adjustment of these molecules is achieved by modifying the benzene rings on both sides of the oxazoline. Yellow molecules can be achieved through modification with weak push-pull structural substituents, such as methoxy (M21, λ_{max} = 440 nm). Modification with dimethylamine redshifts the absorption, and modification with nitro on the other side further redshifts the molecule to give a blue color (M22, λ_{max} = 592 nm). In addition to this, other chromophore modifications can also modulate the color of such molecules.

Supplementary figures

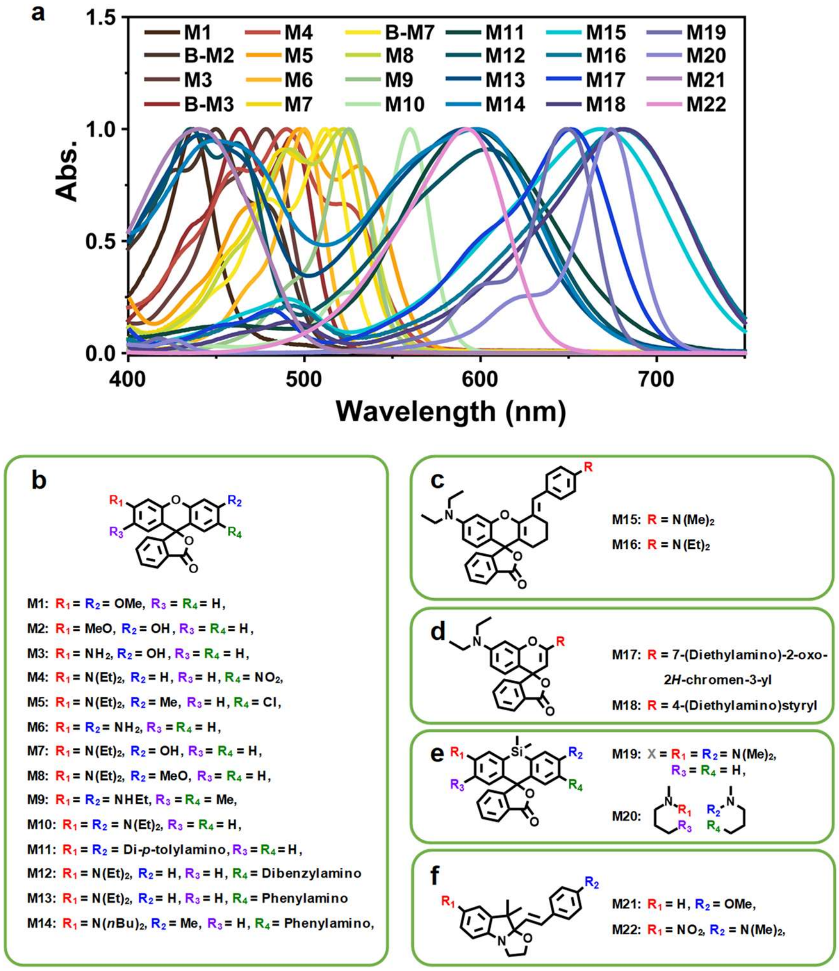

Fig. S1. The normalized absorption spectra (a) of dyes covering the visible region (solvent: MeCN, after acidochromic or basochromic (with prefix “B”) process) and corresponding structure (b-f).

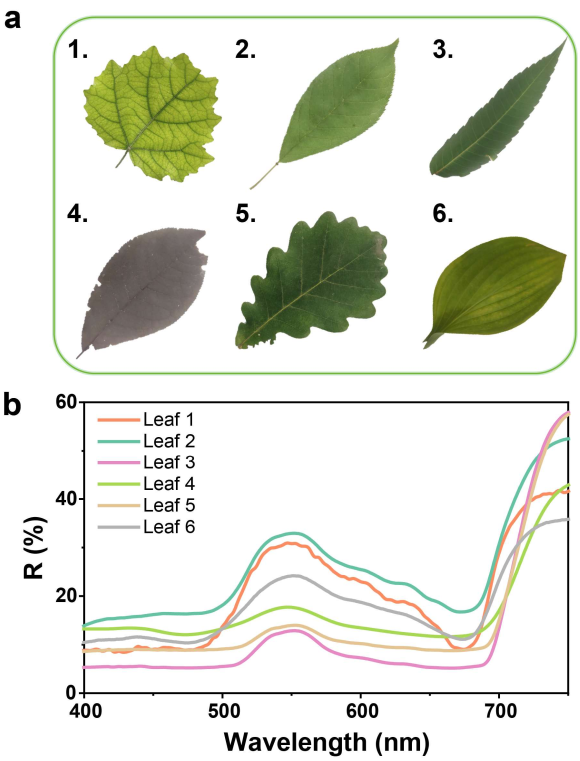

Fig. S2. (a) Photos of common leaves collected from campus, the names of the plants where the leaves collected from are populus alba, elm, rhus typhina, prunus cerasifera 'pissardii', mongolian oak and fragrant plantain lily, corresponding to 1-6 respectively. (b)The visible reflective spectra of leaves collected from campus.

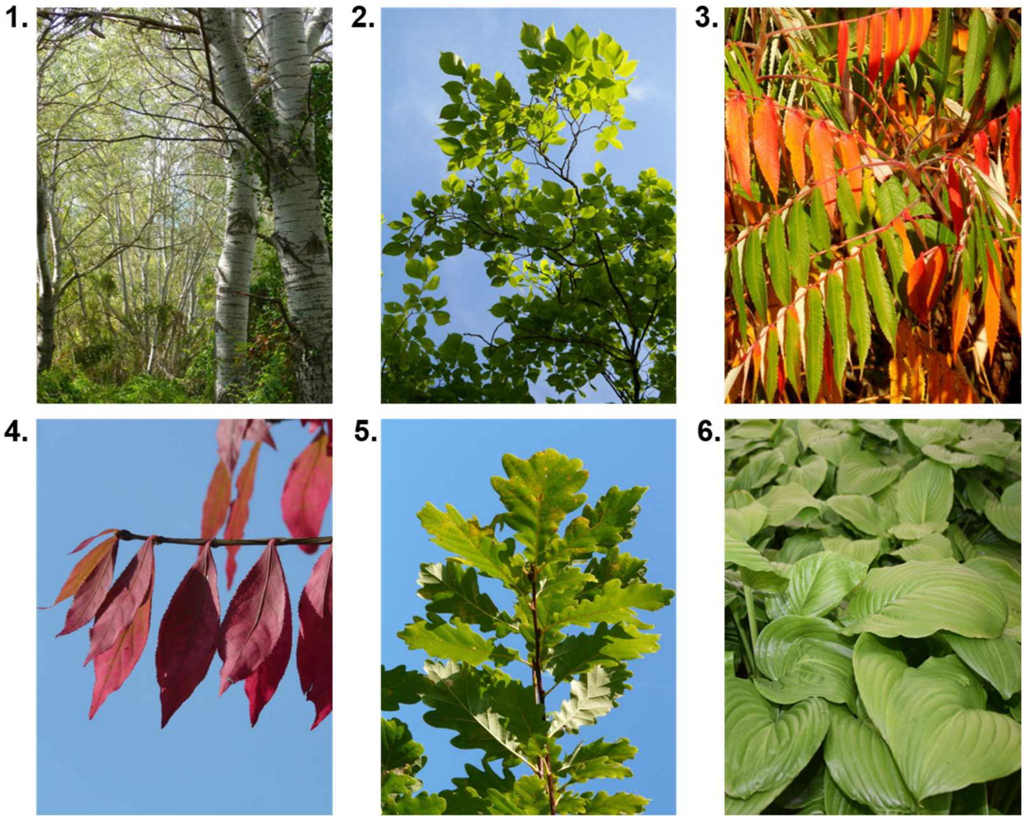

Fig. S3. Photos of the plants offered the common leaves (populus alba, elm, rhus typhina, prunus cerasifera ‘pissardii’, mongolian oak and fragrant plantain lily corresponding to 1-6 respectively, these photos were collected from pixabay.com).

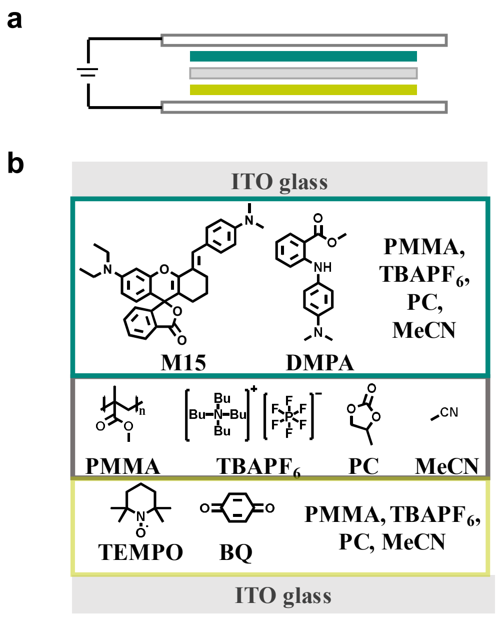

Fig. S4. The device structure (a) and the composition of each layer (b) (top to bottom: ITO (indium tin oxide) glass, electrochromic layer, ion conducting layer, ion storage layer, ITO glass).

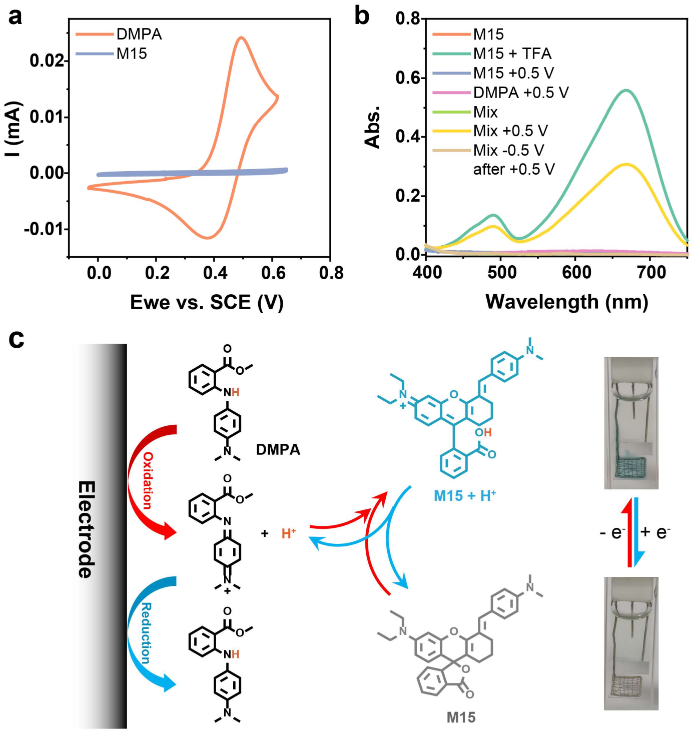

Fig. S5. (a) The cyclic voltammograms of M15 ($1.0*10^{-3}$ M, in MeCN) and DMPA ($1.0*10^{-3}$ M, in MeCN) respectively. (b) The visible absorption spectra of M15, DMPA and their mixture solution at initial, treated with TFA, or electrical stimulation states. (c) Diagram of the electrochromic mechanism based on M15 and DMPA.

To explore the electrochromic mechanism of these dyes using electroacid method, spectroelectrochemistry was performed to observe spectral changes, and here M15 was taken as an example. Firstly, the oxidative peak of DMPA was +0.45 V (vs. SCE, Fig. S5a), and there was no sign of M15 being oxidized at this potential, which guaranteed the oxidation taken place at DMPA instead of dyes. The solutions of M15, DMPA (methyl 2-((4-(dimethylamino)phenyl)amino)benzoate) as electroacid and their mixture were colorless (Fig. S5b), while only their mixture solution showed cyan color under +0.5 V (Fig. S5b yellow line). Besides, the color and absorption peak didn't show in their separate solutions treated with +0.5 V (Fig. S5b). This emerging color and absorption peak were similar to that of M15 treated with TFA (trifluoroacetic acid) (Fig. S5b, cyan line, M15 + TFA), which indicated that the color may due to the generation of the protonated M15. And the color of the mixture solution recovered to the initial state after the stimulation of reversible voltage (Fig. S5b brown line). The results above indicated

that the mixture of M15 and DMPA exhibited electrochromic property. The oxidative process of DMPA could release proton to M15 and the following reductive process could capture proton back from M15, along with the color change, as illustrated in Fig. S5c. DMPA as electroacid and BQ (1,4-benzoquinone) as electrobase could cooperate with all the acido/basochromic dyes in Fig. S1 to happen electrochromism, thus the full visible region electrochromism was realized.

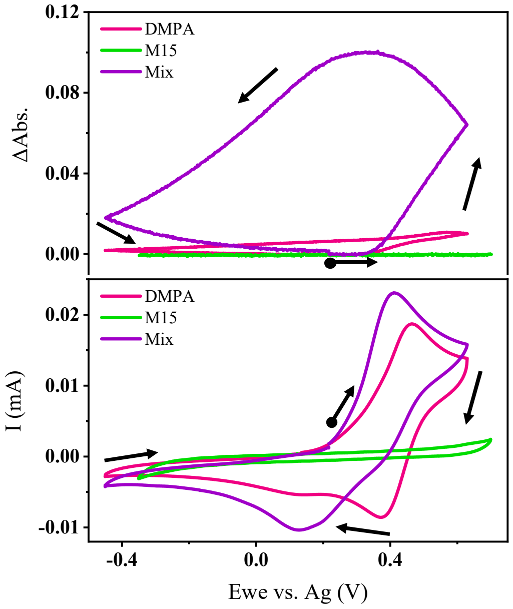

Fig. S6. In-situ absorption change at 668 nm (top) and cyclic voltammogram (bottom) of M15, DMPA and their mixture solution ($1.0*10^{-4}$ M, in MeCN) on platinum net as working electrode (air as the reference).

The cyclic voltammetry of mixture solution was detected in-situ by absorption spectrometer. In Fig. S6, the intensity of absorbance at 668 nm increased gradually with the voltage scanning over the oxidative potential of DMPA. While the voltage scanned back to below the reductive potential of oxidation product of DMPA, the absorption decreased and the solution became colorless gradually. And the coloring/bleaching process could not be seen in their separate solution. These results meant that the electrochromic process was dependent on the oxidation/reduction of DMPA, and the oxidative process of DMPA can reversibly release proton to M15, along with the coloring process.

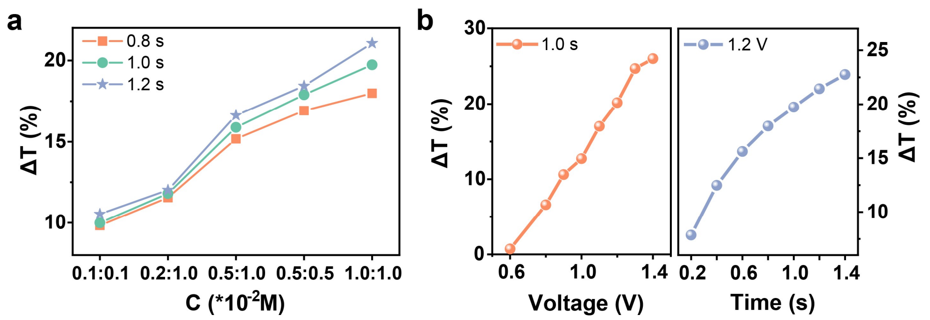

Fig. S7. (a) The transmittance change at 668 nm of devices fabricated with different electrochromic layer solution containing different concentration of M15 : DMPA treated with +1.2 V. (b) The transmittance change at 668 nm of the device applied different voltage for 1.0 s (left) and applied +1.2 V for different time (right).

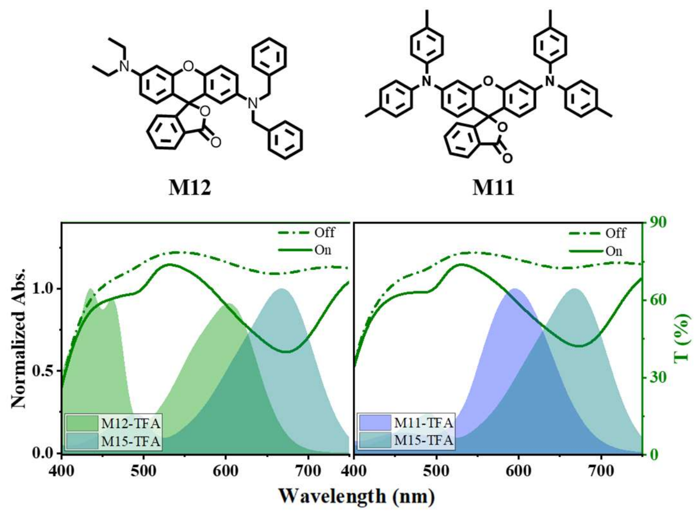

Fig. S8. The transmittance spectra of device made with "DMPA + M12 (left)/ M11 (right) + M15" (EC (Electrochromic layer) solution: M15: $2*10^{-3}$ M, M12/M11 and DMPA: $1*10^{-2}$ M) and the absorption spectra of M12 (green shadow)/ M11 (blue shadow) and M15 (cyan shadow) treated with TFA.

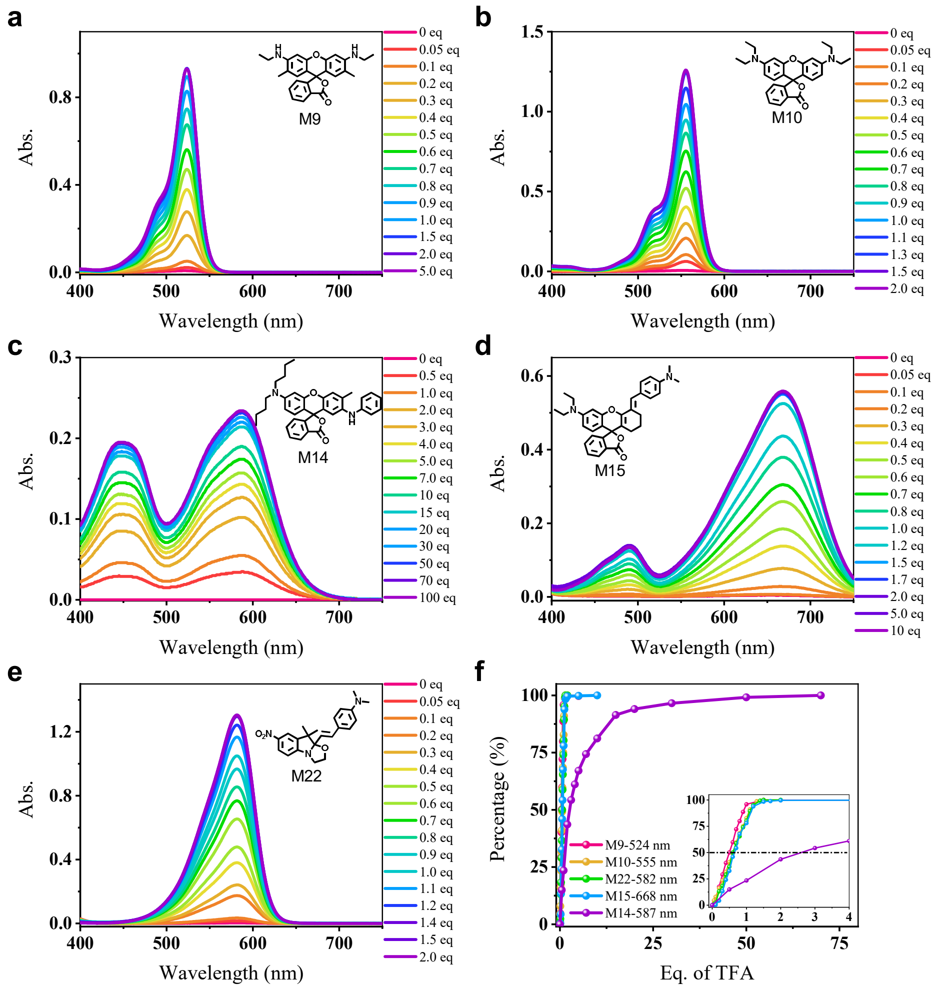

Fig. S9. The absorption spectra of (a) M9, (b) M10, (c) M14, (d) M15, (e) M22 treated with different equivalent of TFA respectively (concentration of dye: $1.0*10^{-5}$ M, in MeCN, room temperature). (f) The coloration percentage of dyes versus the equivalent of TFA.

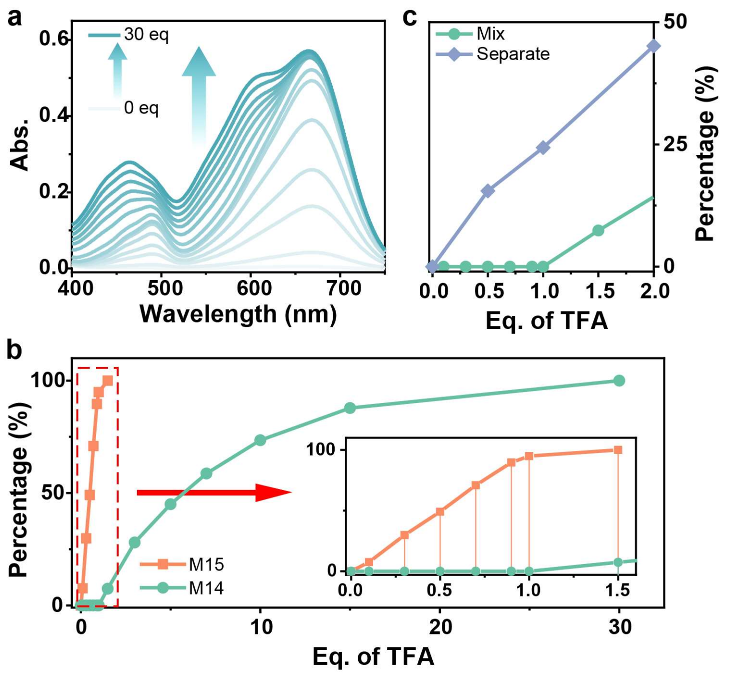

Fig. S10. (a) The absorption spectra of mixture solution of M14 and M15 treated with different equivalent of TFA (concentration of M14 and M15: $1.0*10^{-5}$ M, in MeCN). (b) The coloration percentage of M14 and M15 in their mixture solution. (c) The coloration percentage of M14 in mixture solution with M15 (green line with dot) and M14's own solution (purple line with diamond) versus the equivalent of TFA.

In order to analyze this unexpected incompatible phenomenon, the sensitivity of M14, M15 and several other dyes to proton was measured using TFA as the source of proton. To reach their 50% maximum coloration, 0.66 equivalent (for M15) and 2.58 equivalent (for M14) TFA was needed (Fig. S9). The big difference made their mixture solution appeared the coloration of M15 first, and M14 started to color after M15 colored to 100% as the equivalent of TFA increasing (Fig. S10a, b). There exists the coloration sequence in the mixture of M14 and M15, M14 colored to 25% in its individual solution as treated with 1.0 equivalent of TFA (Fig. S10c), yet 0% in the mixture solution with M15 as treated with 1.0 equivalent of TFA (Fig.S10c). Therefore, the difference of sensitivity of dyes to proton made them incompatible to working together in the same layer.

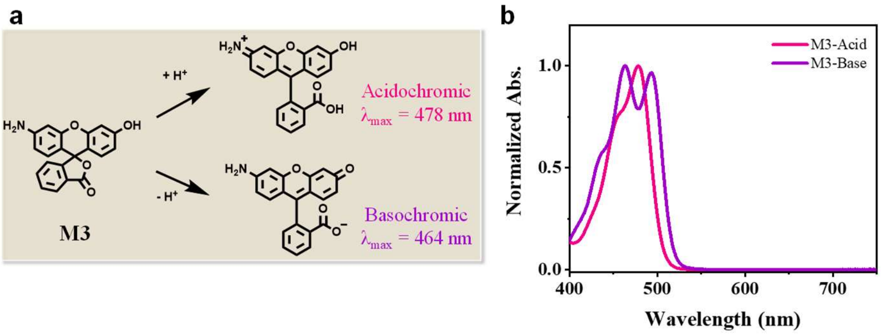

Fig. S11. (a) The structure change of M3 of acido/basochromic process. (b) The absorption spectra of M3 treated with acid or base.

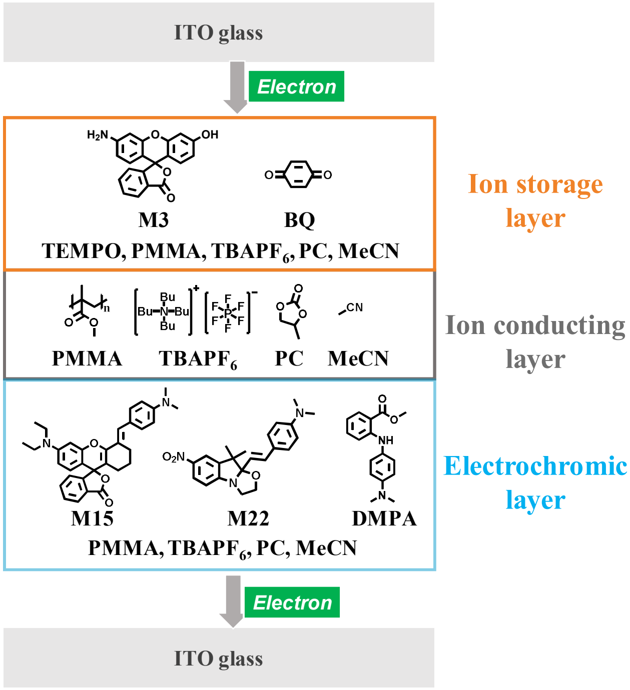

Fig. S12. The schematic of double-layer electrochromism device.

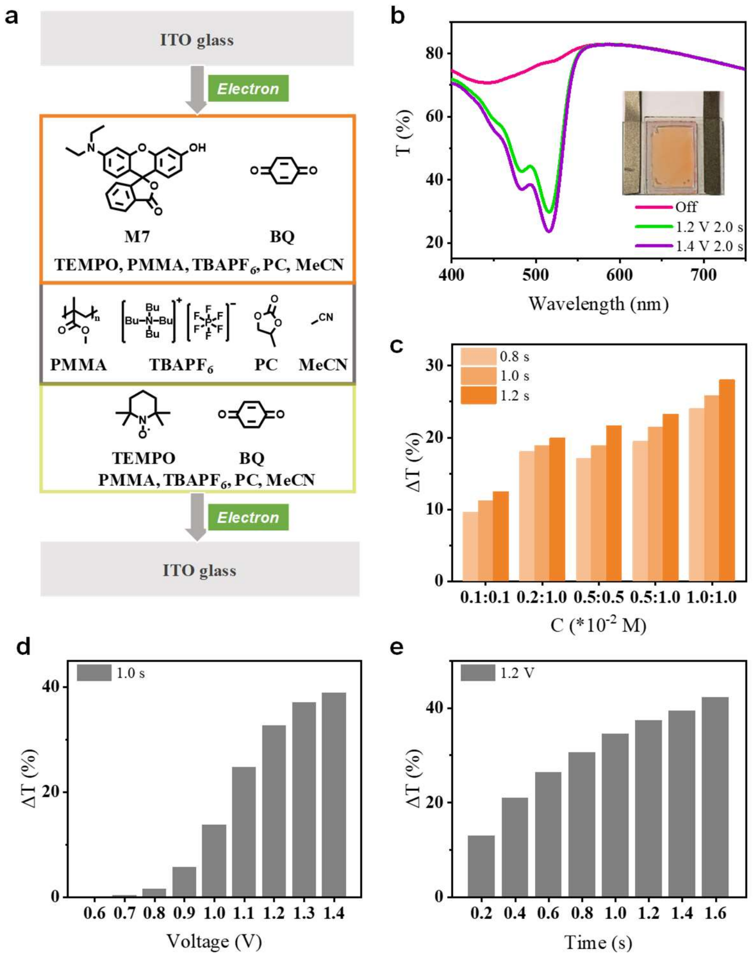

Fig. S13. Electrochromic properties of device made of M7 and BQ. (a) The device structure (top to bottom: ITO glass, ion storage layer, ion conducting layer, electrochromic layer, ITO glass). (b) The transmittance spectra of device before and after applied voltage for 2.0 s. (c) The transmittance change at 516 nm of devices made of M7 : BQ with different concentration applied +1.2 V. (d) The transmittance change at 516 nm of device applied different voltage for 1.0 s. (e) The transmittance change at 516 nm applied +1.2 V for different time.

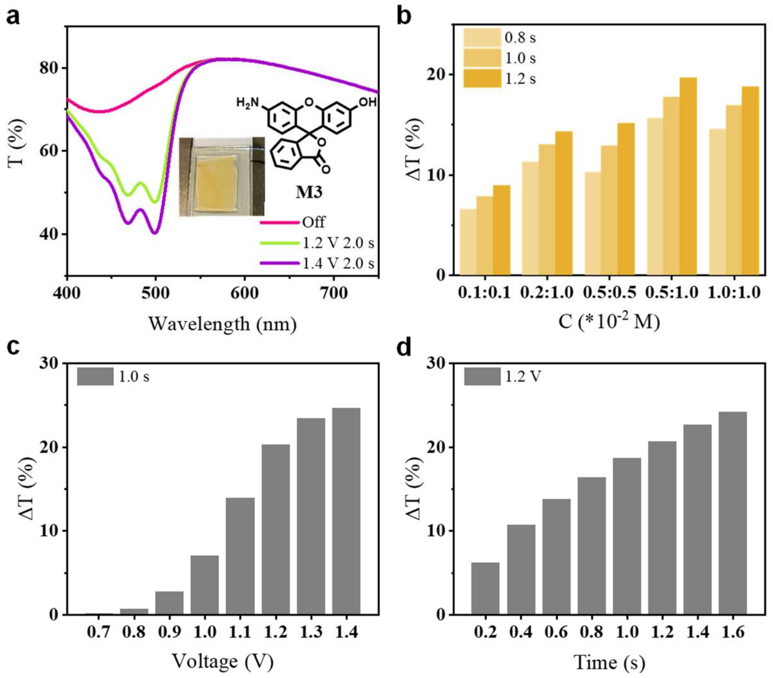

Fig. S14. (a) The transmittance spectra of device made with M3 and BQ before and after applied voltage for 2.0 s. (b) The transmittance change at 499 nm of devices made of M3 : BQ with different concentration applied +1.2 V. (c) The transmittance change at 499 nm of device applied different voltage for 1.0 s. (d) The transmittance change at 499 nm applied +1.2 V for different time.

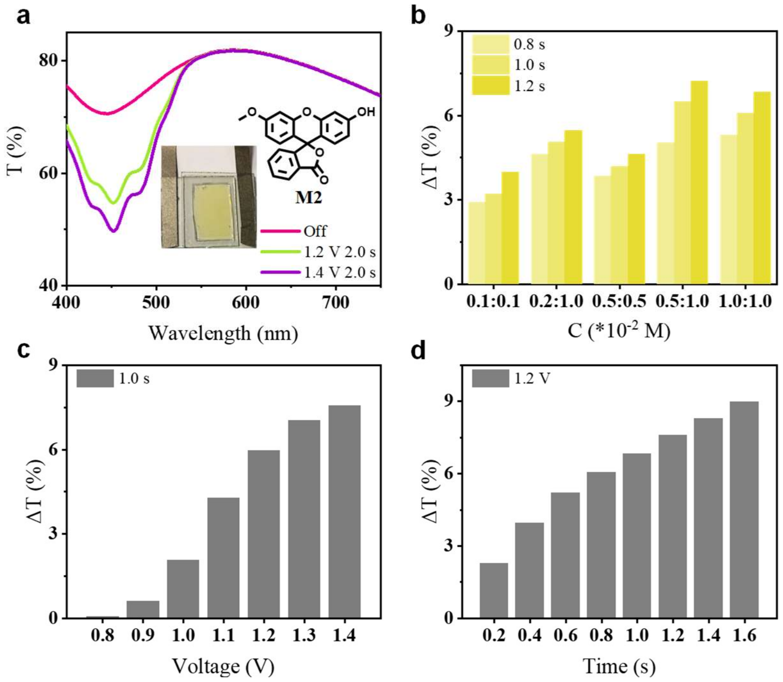

Fig. S15. (a) The transmittance spectra of device made with M2 and BQ before and after applied voltage for 2.0 s. (b) The transmittance change at 462 nm of devices made of M2 : BQ with different

concentration applied +1.2 V. (c) The transmittance change at 462 nm of device applied different voltage for 1.0 s. (d) The transmittance change at 462 nm applied +1.2 V for different time.

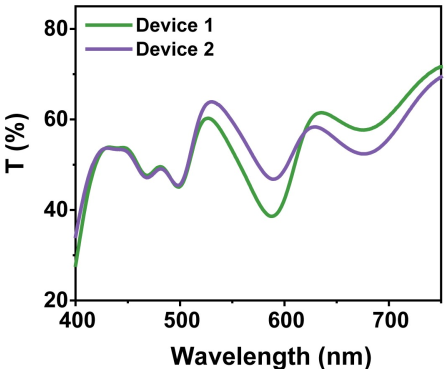

Fig. S16. Devices with different concentration of dyes treated with +1.4 V for 2 s (Device 1: M15: $2*10^{-3}$ M, M22: $1*10^{-2}$ M, DMPA: $1*10^{-2}$ M, M3: $5*10^{-3}$ M, device 2: M15: $2*10^{-3}$ M, M22: $5*10^{-3}$ M, DMPA: $1*10^{-2}$ M, M3: $5*10^{-3}$ M).

For the device with several kinds of dyes, the absorption intensity could be regulated by the concentration of corresponding dye, bigger concentration leads to stronger absorption intensity at its wavelength, like Fig. S16 shows, the concentration of M22 in device 1 was bigger than device 2, which led to lower transmittance at M22's λ_{max} (592 nm) after the same electrical treatment.

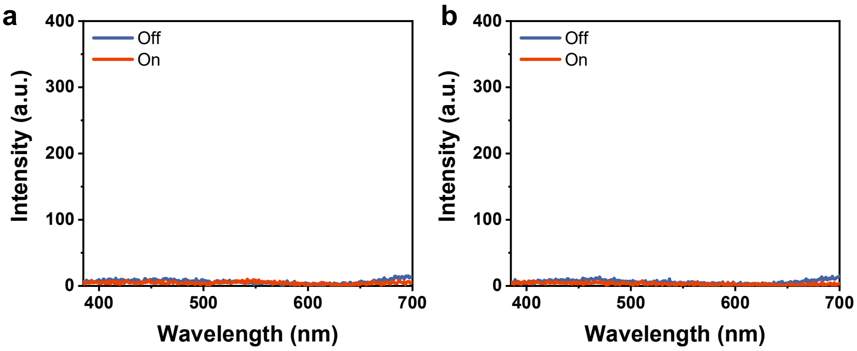

Fig. S17. (a) The fluorescence spectra of the device used to simulate leaf 2 on its "off" state and "on" state (+1.4 V 2.0 s) (excitation light: 365 nm, slit width (3, 3)). (b) The fluorescence spectra of the device used to simulate leaf 4 on its "off" state and "on" state (+1.5 V 3.0 s) (excitation light: 365 nm, slit width (3, 3)).

The emission of dyes has very little interference with the color the device. Firstly, except for M9 and M10, the fluorescence efficiency of the dyes is rather low. Secondly, because of the high concentration of dyes in device, the emission was quenched seriously, known as ACQ (aggregation-

caused quenching). The emission spectra of the device were recorded by Shimadzu RF-5301 PC spectrofluorophotometer with the excitation light of 365 nm and the slit width (3, 3), as Fig. S17 shows. The device showed nearly no fluorescence at “off” and “on” states.

Therefore, we think the interference of emission with the color of the device is negligible.

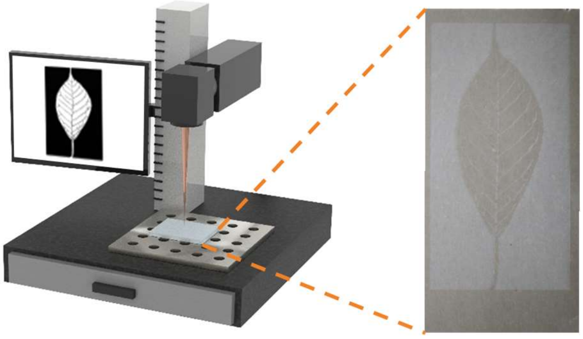

Fig. S18. The schematic of laser etching process and the photo of etched ITO electrode glass.

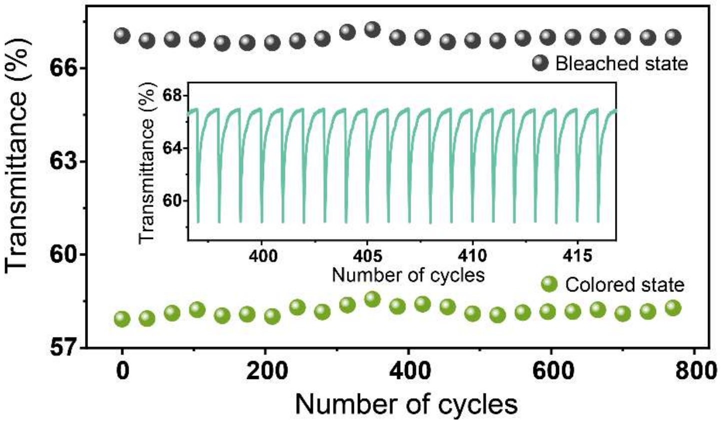

Fig. S19. The transmittance at 498 nm of the device under coloring-bleaching (+1.2 V for 1.0 s, power off for 0.6 s, -0.85 V for 11 s, power off for 22 s) cycles for 780 times.

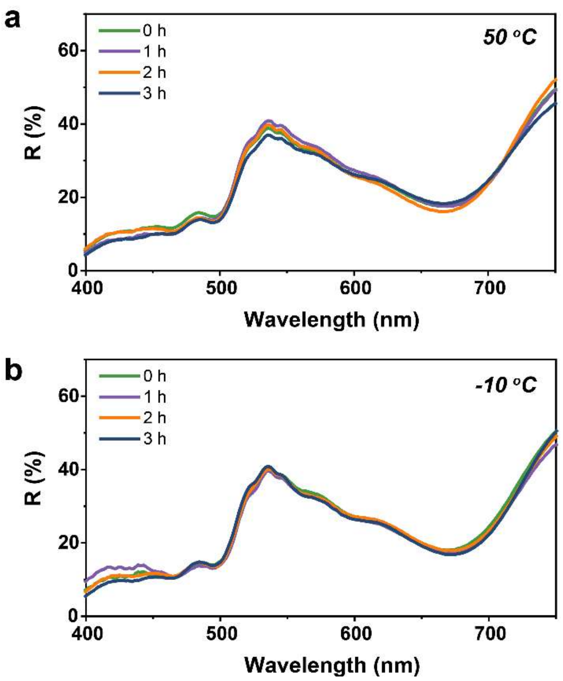

Fig. S20. The reflectance spectra of the device to simulate leaf 2 under "on" state after treated with (a) 50 °C or (b) -10 °C for 0-3 h.

Weather resistance of the device was tested, the device maintained the ability to simulate leaf 2, but the device needed longer electrical stimulation to simulate leaf 2 under 50 °C or -10 °C: as-prepared (0 h) needed +1.4 V for 2.0 s, after 50 °C for 1 h needed +1.4 V for 2.0 s, after 50 °C for 2 h needed +1.4 V for 2.2 s, after 50 °C for 3 h needed +1.4 V for 2.5 s; after -10 °C for 1 h needed +1.4 V for 2.5 s, after -10 °C for 2 h needed +1.4 V for 3.5 s, after -10 °C for 3 h needed +1.4 V for 4.5 s.

Supplementary functions

The functions to fit the spectrum of every dye are listed as below.

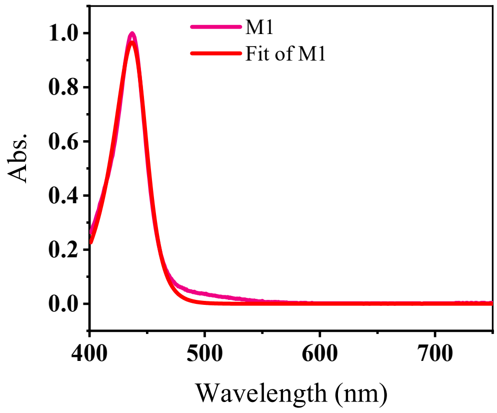

Model	BiHill
Function	y = Pm/(1+(Ka/x)^Ha)/(1+(x/Ki)^Hi);
f(x)	Abs.
Pm	25072.28762 ± 1.76242E7
Ka	699.21811 ± 23542.49152
Ki	442.32653 ± 0.2368
Ha	20.88533 ± 0.58068
Hi	72.3587 ± 1.34759
Reduced Chi-Sqr	2.31E-04

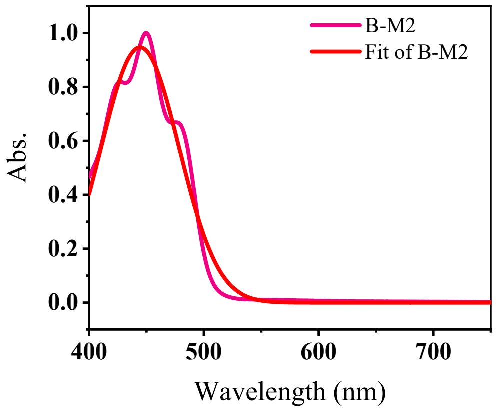

Model	GaussAmp
Function	y=y0+A*exp(-0.5*((x-xc)/w)^2)
f(x)	Abs.
y0	-4.13097E-4 ± 0.00216
xc	444.47228 ± 0.26611
w	34.01226 ± 0.31194
A	0.94707 ± 0.00604
Reduced Chi-Sqr	1.25E-03

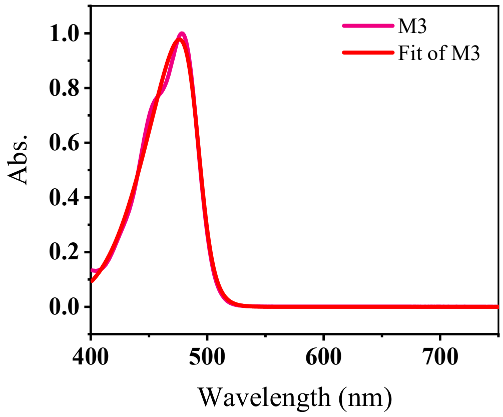

Model	BiHill
Function	y = Pm/(1+(Ka/x)^Ha)/(1+(x/Ki)^Hi);
f(x)	Abs.
Pm	1.48671 ± 0.0639
Ka	455.93461 ± 1.10437
Ki	491.71797 ± 0.62885
Ha	20.96071 ± 0.55862
Hi	76.71647 ± 3.36604
Reduced Chi-Sqr	2.03E-04

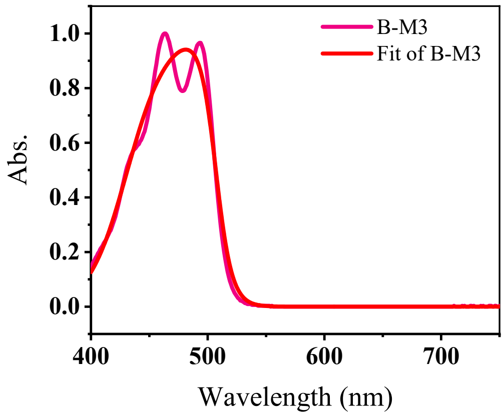

Model	BiHill
Function	y = Pm/(1+(Ka/x)^Ha)/(1+(x/Ki)^Hi);
f(x)	Abs.
Pm	1.02344 ± 0.1191
Ka	432.1029 ± 3.45287
Ki	506.8858 ± 1.62705
Ha	25.39615 ± 1.71747
Hi	73.81401 ± 8.26564
Reduced Chi-Sqr	1.44E-03

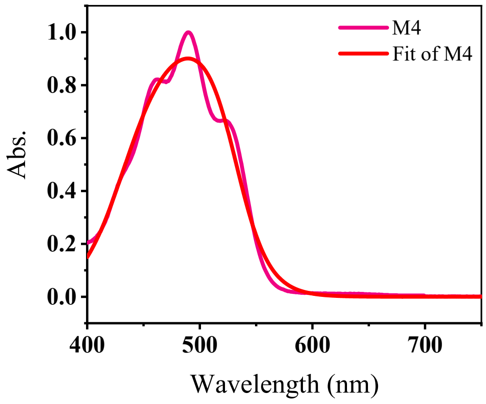

Model	BiHill
Function	y = Pm/(1+(Ka/x)^Ha)/(1+(x/Ki)^Hi);
f(x)	Abs.
Pm	1.01436 ± 0.03804
Ka	434.32825 ± 1.62187
Ki	532.40675 ± 1.17342
Ha	21.27163 ± 0.94411
Hi	37.14122 ± 1.63588
Reduced Chi-Sqr	1.19E-03

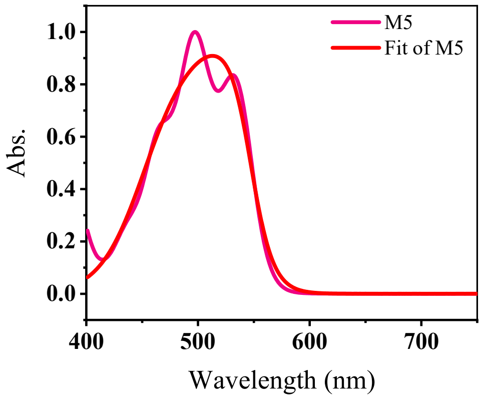

Model	BiHill
Function	y = Pm/(1+(Ka/x)^Ha)/(1+(x/Ki)^Hi);
f(x)	Abs.
Pm	1.00237 ± 0.08302
Ka	454.53092 ± 3.14033
Ki	547.38227 ± 1.68483
Ha	21.56816 ± 1.10061
Hi	55.28869 ± 4.72917
Reduced Chi-Sqr	1.62E-03

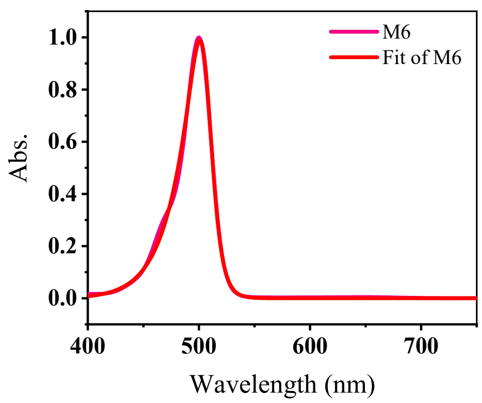

Model	BiHill
Function	y = Pm/(1+(Ka/x)^Ha)/(1+(x/Ki)^Hi);
f(x)	Abs.
Pm	5590.02799 ± 931646.92887
Ka	726.31723 ± 5355.76398
Ki	507.12898 ± 0.17448
Ha	22.62221 ± 0.35733
Hi	107.27319 ± 0.92893
Reduced Chi-Sqr	1.07E-04

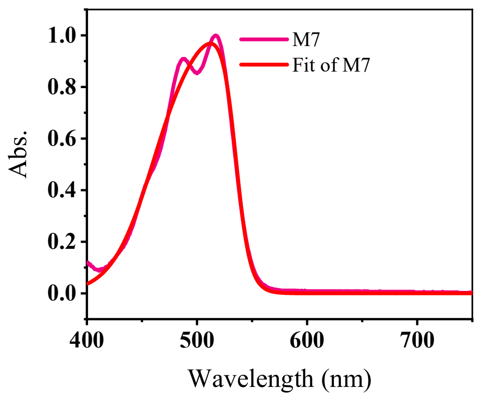

Model	BiHill
Function	y = Pm/(1+(Ka/x)^Ha)/(1+(x/Ki)^Hi);
f(x)	Abs.
Pm	1.08521 ± 0.01295
Ka	462.60921 ± 0.67257
Ki	535.27338 ± 0.20965
Ha	23.17757 ± 0.4803
Hi	84.27869 ± 2.03414
Reduced Chi-Sqr	5.49E-04

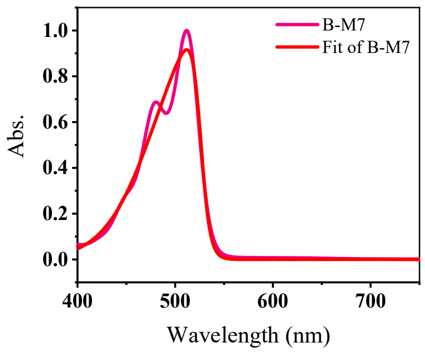

Model	BiHill
Function	y = Pm/(1+(Ka/x)^Ha)/(1+(x/Ki)^Hi);
f(x)	Abs.
Pm	1.33473 ± 0.06068
Ka	484.95188 ± 2.78294
Ki	525.87551 ± 0.2601
Ha	17.25479 ± 0.58674
Hi	114.11282 ± 4.03114
Reduced Chi-Sqr	8.29E-04

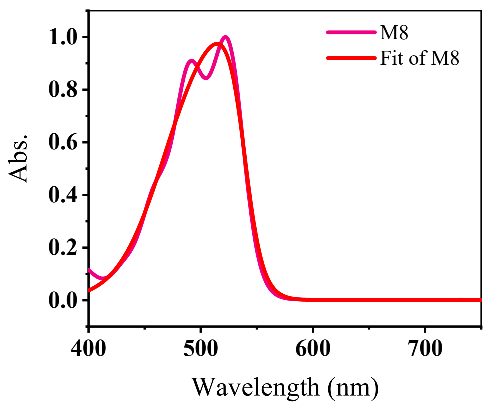

Model	BiHill
Function	y = Pm/(1+(Ka/x)^Ha)/(1+(x/Ki)^Hi);
f(x)	Abs.
Pm	1.16471 ± 0.11689
Ka	470.07913 ± 2.61634
Ki	539.06663 ± 1.61349
Ha	21.17911 ± 0.51136
Hi	68.03864 ± 5.95082
Reduced Chi-Sqr	8.02E-04

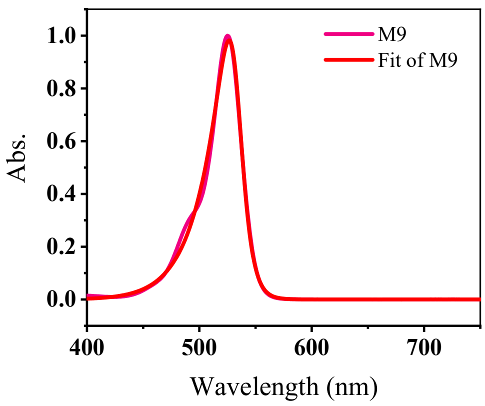

Model	BiHill
Function	y = Pm/(1+(Ka/x)^Ha)/(1+(x/Ki)^Hi);
f(x)	Abs.
Pm	51191.08021 ± 9.75343E7
Ka	852.99429 ± 73806.46723
Ki	533.16882 ± 0.23087
Ha	22.02248 ± 0.43186
Hi	105.23492 ± 1.14448
Reduced Chi-Sqr	1.78E-04

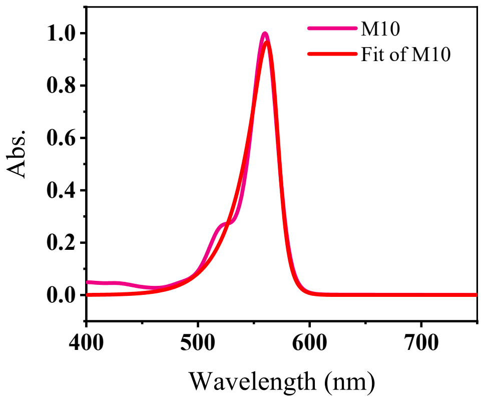

Model	BiHill
Function	y = Pm/(1+(Ka/x)^Ha)/(1+(x/Ki)^Hi);
f(x)	Abs.
Pm	139885.25193 ± 7.93287E8
Ka	935.12546 ± 231621.03534
Ki	568.34376 ± 0.29606
Ha	22.8966 ± 0.57798
Hi	121.98557 ± 2.42633
Reduced Chi-Sqr	6.59E-04

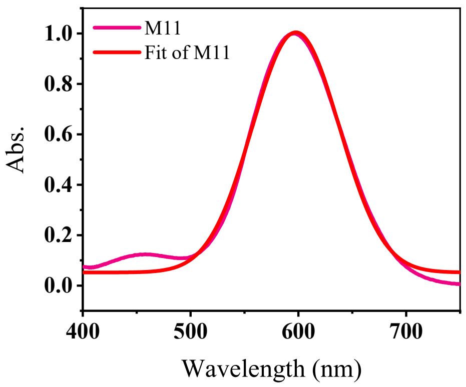

Model	Gauss
Function	y=y0 + (A/(w*sqrt(pi/2)))*exp(-2*((x-xc)/w)^2)
f(x)	Abs.
y0	0.05205 ± 0.00253
xc	597.62113 ± 0.24456
w	80.68766 ± 0.57572
A	96.31639 ± 0.74253
Reduced Chi-Sqr	1.19E-03

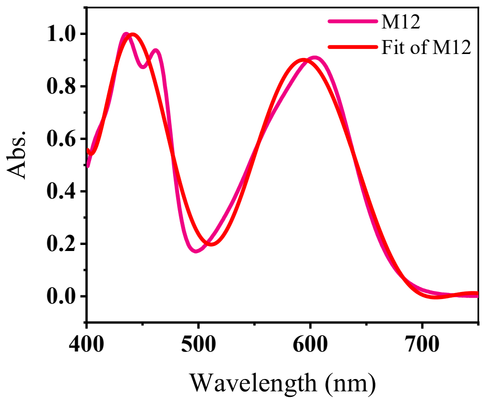

Model	Poly
Function	y = a0+a1*x+a2*x^2+a3*x^3+a4*x^4+a5*x^5 +a6*x^6+a7*x^7+a8*x^8+a9*x^9;
f(x)	Abs.
a0	309692.73133
a1	-4804.12637
a2	32.7951
a3	-0.12932
a4	3.24701E-4
a5	-5.38451E-7
a6	5.89906E-10
a7	-4.11848E-13
a8	1.66325E-16
a9	-2.96141E-20
Reduced Chi-Sqr	0.00243

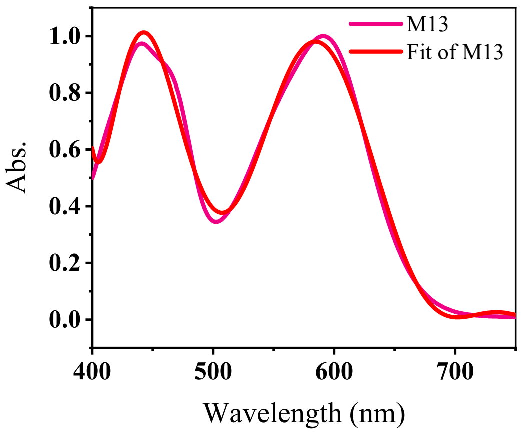

Model	Poly
Function	y = a0+a1*x+a2*x^2+a3*x^3+a4*x^4+a5*x^5+a6*x^6+a7*x^7+a8*x^8+a9*x^9;
f(x)	Abs.
a0	363482.82451 ± 1271.20188
a1	-5675.16692 ± 16.23131
a2	39.01417 ± 0.08629
a3	-0.15501 ± 2.40772E-4
a4	3.92344E-4 ± 3.52939E-7
a5	-6.56178E-7 ± 2.69861E-10
a6	7.25326E-10 ± 5.62969E-13
a7	-5.11126E-13 ± 7.65367E-16
a8	2.08419E-16 ± 4.77329E-19
a9	-3.748E-20 ± 1.17277E-22
Reduced Chi-Sqr	8.20E-04

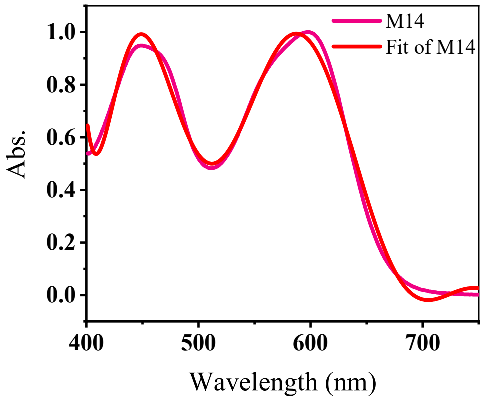

Model	Poly
Function	y = a0+a1*x+a2*x^2+a3*x^3+a4*x^4+a5*x^5+a6*x^6+a7*x^7+a8*x^8+a9*x^9;
f(x)	Abs.
a0	298952.72341
a1	-4603.03651
a2	31.19288
a3	-0.12211
a4	3.0439E-4
a5	-5.0109E-7
a6	5.44894E-10
a7	-3.77513E-13
a8	1.51249E-16
a9	-2.67066E-20
Reduced Chi-Sqr	9.27E-04

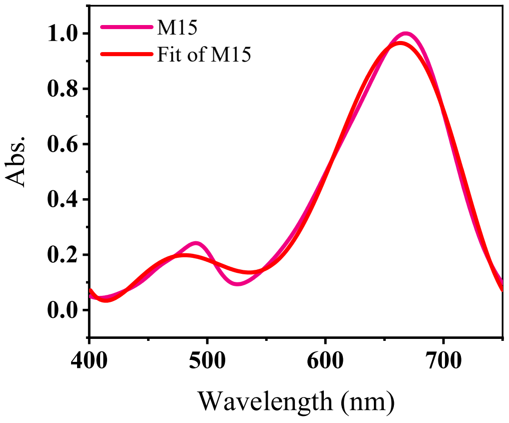

Model	Poly
Function	y = a0+a1*x+a2*x^2+a3*x^3+a4*x^4+a5*x^5+a6*x^6+a7*x^7+a8*x^8+a9*x^9;
f(x)	Abs.
a0	9601.61994
a1	-163.43004
a2	1.25598
a3	-0.00569
a4	1.66504E-5
a5	-3.24623E-8
a6	4.19794E-11
a7	-3.46041E-14
a8	1.64597E-17
a9	-3.43685E-21
Reduced Chi-Sqr	7.05E-04

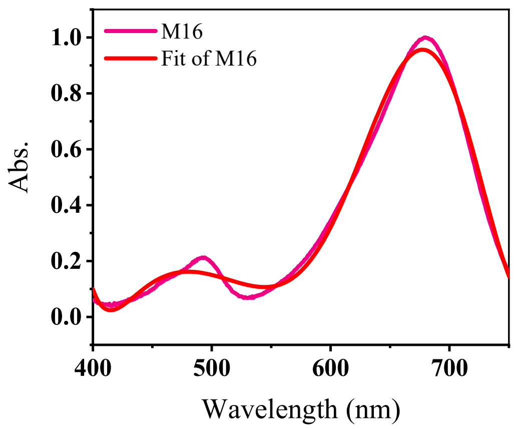

Model	Poly
Function	y = a0+a1*x+a2*x^2+a3*x^3+a4*x^4+a5*x^5+a6*x^6+a7*x^7+a8*x^8+a9*x^9;
f(x)	Abs.
a0	80311.56024 ± 1200.48046
a1	-1306.5975 ± 15.3283
a2	9.40278 ± 0.08149
a3	-0.03928 ± 2.27377E-4
a4	1.04903E-4 ± 3.33304E-7
a5	-1.85745E-7 ± 2.54847E-10
a6	2.17942E-10 ± 5.31649E-13
a7	-1.63346E-13 ± 7.22787E-16
a8	7.09371E-17 ± 4.50773E-19
a9	-1.35958E-20 ± 1.10753E-22
Reduced Chi-Sqr	7.31E-04

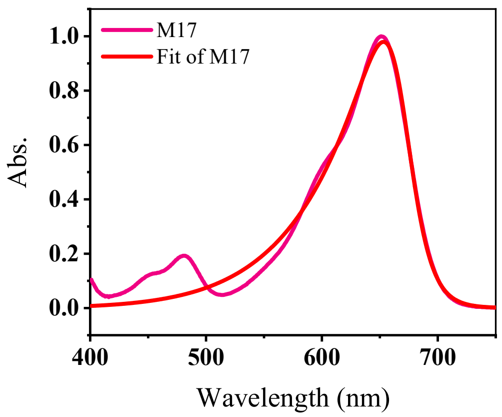

Model	BiHill
Function	y = Pm/(1+(Ka/x)^Ha)/(1+(x/Ki)^Hi);
f(x)	Abs.
Pm	59030.31268 ± 3.80637E8
Ka	1881.57413 ± 1.18369E6
Ki	669.82276 ± 1.10447
Ha	10.25097 ± 0.55384
Hi	69.2744 ± 1.96605
Reduced Chi-Sqr	2.47E-03

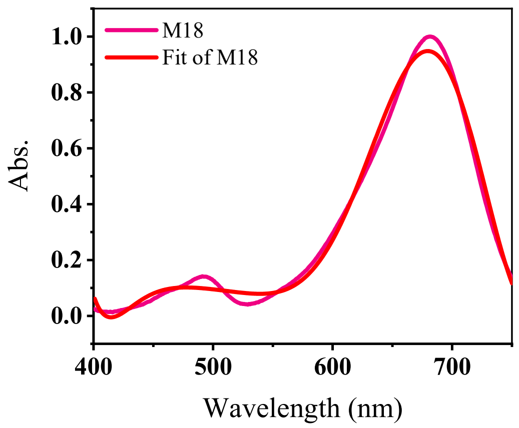

Model	Poly
Function	y = a0+a1*x+a2*x^2+a3*x^3+a4*x^4+a5*x^5+a6*x^6+a7*x^7+a8*x^8+a9*x^9;
f(x)	Abs.
a0	117863.85176
a1	-1916.81946
a2	13.77352
a3	-0.05739
a4	1.52741E-4
a5	-2.69293E-7
a6	3.1442E-10
a7	-2.34386E-13
a8	1.01207E-16
a9	-1.92835E-20
Reduced Chi-Sqr	7.73E-04

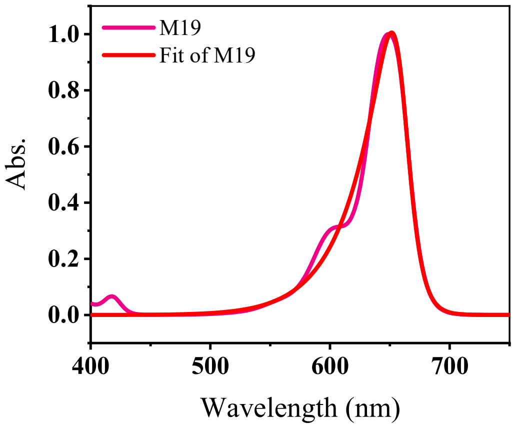

Model	BiHill
Function	y = Pm/(1+(Ka/x)^Ha)/(1+(x/Ki)^Hi);
f(x)	Abs.
Pm	9940.14174 ± 7.02816E6
Ka	1027.56644 ± 36816.33258
Ki	660.54067 ± 0.51216
Ha	19.74463 ± 0.72213
Hi	108.73312 ± 1.98297
Reduced Chi-Sqr	7.44E-04

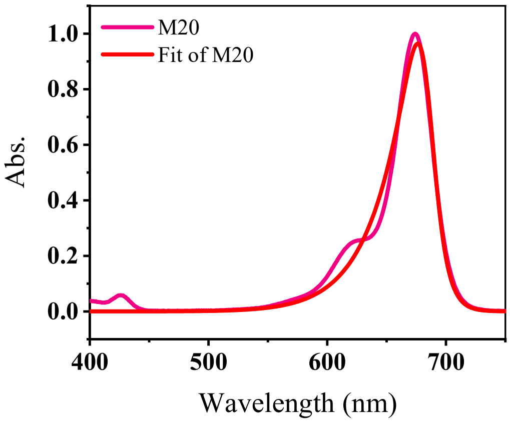

Model	BiHill
Function	y = Pm/(1+(Ka/x)^Ha)/(1+(x/Ki)^Hi);
f(x)	Abs.
Pm	20389.97848 ± 3.55111E7
Ka	1059.40721 ± 85001.41823
Ki	684.84174 ± 0.5949
Ha	21.71201 ± 0.92458
Hi	113.84578 ± 2.45749
Reduced Chi-Sqr	9.22E-04

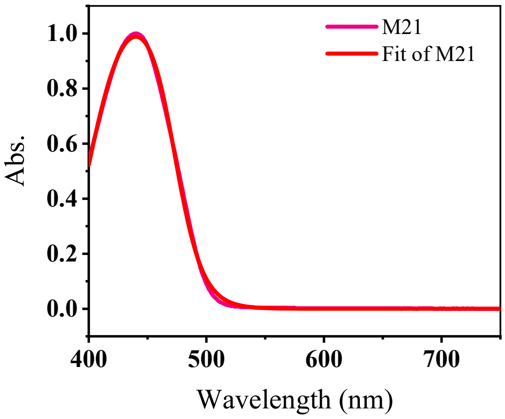

Model	BiHill
Function	y = Pm/(1+(Ka/x)^Ha)/(1+(x/Ki)^Hi);
f(x)	Abs.
Pm	1.11903 ± 0.00829
Ka	401.85895 ± 0.19532
Ki	473.32453 ± 0.19309
Ha	28.35673 ± 0.66642
Hi	40.55276 ± 0.33213
Reduced Chi-Sqr	6.34E-05

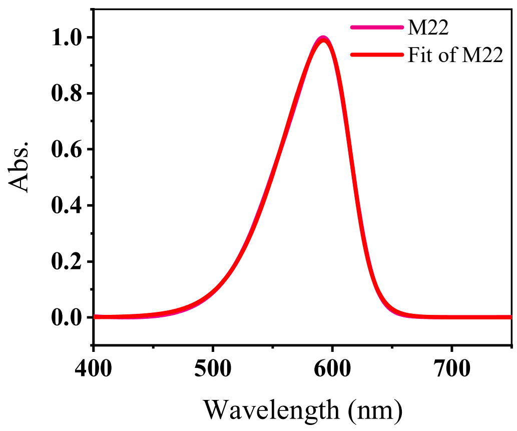

Model	BiHill
Function	y = Pm/(1+(Ka/x)^Ha)/(1+(x/Ki)^Hi);
f(x)	Abs.
Pm	1.50175 ± 0.01107
Ka	566.91294 ± 0.40056
Ki	613.54077 ± 0.09224
Ha	21.79599 ± 0.10369
Hi	66.41559 ± 0.27795
Reduced Chi-Sqr	2.09E-05

Synthesis methods

Synthesis of DMPA[1]

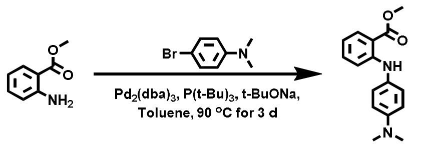

Methyl anthranilate (0.5 mmol, 75.6 mg), 4-bromo-*N*,*N*-dimethylaniline (0.55 mmol, 110.0 mg) and sodium tert-butoxide (1.0 mmol, 96.1 mg) were placed in the reaction flask under nitrogen flow, then dry toluene (20 mL) were added as solvent. The flask was cooled by liquid nitrogen, then the air in the reaction system was replaced to nitrogen. Under the protection of nitrogen, tris(dibenzylideneacetone)dipalladium (0.01 mmol, 9.16 mg) and tri-tert-butylphosphine (0.03 mmol) were added to the flask, then the air in the reaction system was replaced to nitrogen again. The reaction system was then allowed to room temperature slowly, stirred for 30 min, then the reaction was carried out at 90 °C for 3 d. Toluene was removed under reduced pressure, then column chromatography was performed using PE/EtOAc as eluent to obtain the product as light yellow solid (yield = 74.4%). ^{1}H NMR (500 MHz, DMSO-*d*$_6$) δ (TMS, ppm) 9.09 (s, 1H), 7.85 (d, *J* = 7.4 Hz, 1H), 7.31 (t, *J* = 7.4 Hz, 1H), 7.08 (d, *J* = 8.6 Hz, 2H), 6.86 (d, *J* = 8.5 Hz, 1H), 6.77 (d, *J* = 8.7 Hz, 2H), 6.66 (t, *J* = 7.4 Hz, 1H), 3.84 (s, 3H), 2.89 (s, 6H). ^{13}C NMR (126 MHz, DMSO-*d*$_6$) δ (DMSO 39.6 ppm) 168.29, 149.50, 148.17, 134.53, 131.24, 128.77, 125.67, 115.92, 113.41, 113.00, 109.92, 51.84, 40.46. LC-HRMS (ESI) calcd. for $C_{16}H_{19}N_2O_2$ [M+H]$^+$: 271.1441, found: 271.1438.

Acquisition Parameter

Source Type	ESI	Ion Polarity	Positive	Set Nebulizer	0.4 Bar
Focus	Active	Set Capillary	4500 V	Set Dry Heater	180 °C
Scan Begin	50 m/z	Set End Plate Offset	-500 V	Set Dry Gas	4.0 l/min
Scan End	3000 m/z	Set Collision Cell RF	150.0 Vpp	Set Divert Valve	Waste

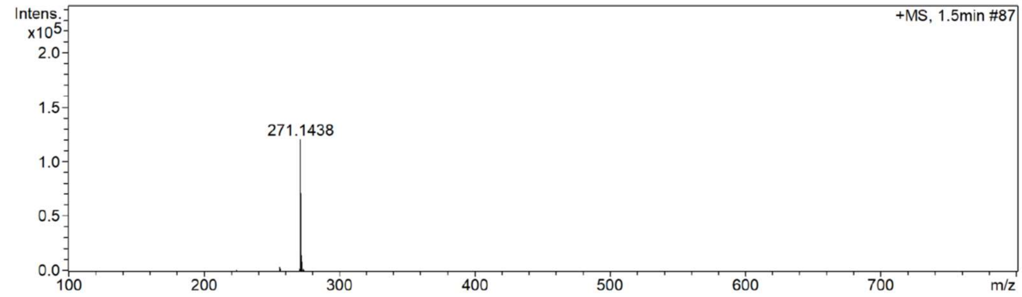

Meas. m/z	#	Formula	m/z	err [ppm]	rdb	N-Rule	e¡¥Conf	mSigma
271.1438								
	1	C 11 H 19 N 4 O 4	271.1401	-13.9	4.5	ok	even	3.92
	2	C 12 H 15 N 8	271.1414	-8.9	9.5	ok	even	18.62
	3	C 16 H 19 N 2 O 2	271.1441	1.0	8.5	ok	even	30.85

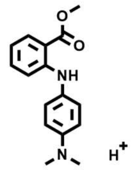

Chemical Formula: $C_{16}H_{19}N_2O_2^+$
Exact Mass: 271.1441
m/z: 271.1441 (100.0%), 272.1475 (17.3%), 273.1508 (1.4%)

Fig. S21. Copy of the mass spectrum report of DMPA.

Synthesis of M15[2,3]

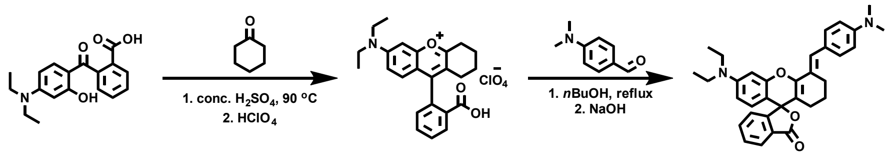

Cyclohexanone (64 mmol, 6.28 g) was added to concentrated H_2SO_4 (70 mL) slowly, then the solution was cooled to 0 °C. 2-(4-Diethylamino-2-hydroxybenzoyl)benzoicacid (32 mmol, 10.03 g) was added to the solution above in portions under violent stirring, then the solution was heated to 90 °C and the reaction was carried for 3 h. After the solution was cooled to room temperature, it was poured to 300 g crushed ice, then $HClO_4$ (7 mL, 70%) was added dropwise under stirring and the precipitate was formed. Let the solution stand for 1 h, then the liquid was decanted out. The precipitate was washed with cold water (100 mL) twice, then the precipitate was dried under reduced pressure. The product was used for next step without further purification.

The product above and 4-(dimethylamino)benzaldehyde (38.4 mmol, 5.73 g) was placed in a round flask, then n-butanol (150 mL) was added as solvent, the reaction mixture was heated to reflux for 10 h. Butanol was removed under reduced pressure, then 200 mL EtOAc was and 200 mL 1M NaOH solution were added, the mixture was stirred at room temperature for 1 h. The organic phase was separated and the aqueous layer was extracted with DCM and EtOAc twice. The organic layers were

combined and dried over Na_2SO_4. Then the product was separated with column chromatography (PE/DCM/EtOAc=1/1/1, with 0.5% triethylamine, as eluent), the product was obtained as grey solid (yield= 56.3%). ^{1}H NMR (400 MHz, DMSO-d_6) δ 7.93 (d, J = 7.6 Hz, 1H), 7.78 (t, J = 7.5 Hz, 1H), 7.66 (t, J = 7.5 Hz, 1H), 7.36 – 7.26 (m, 4H), 6.74 (d, J = 8.3 Hz, 2H), 6.52 (s, 1H), 6.43 (d, J = 9.0 Hz, 1H), 6.37 (d, J = 8.8 Hz, 1H), 3.35 (q, J = 6.2 Hz, 4H), 2.94 (s, 6H), 2.81 – 2.73 (m, 1H), 2.66 – 2.57 (m, 1H), 1.87 – 1.78 (m, 1H), 1.63 – 1.49 (m, 3H), 1.09 (t, J = 6.8 Hz, 6H). LC-HRMS (ESI) calcd. for $C_{33}H_{34}N_2O_3$ $[M+H]^+$: 507.2642, found: 507.2647.

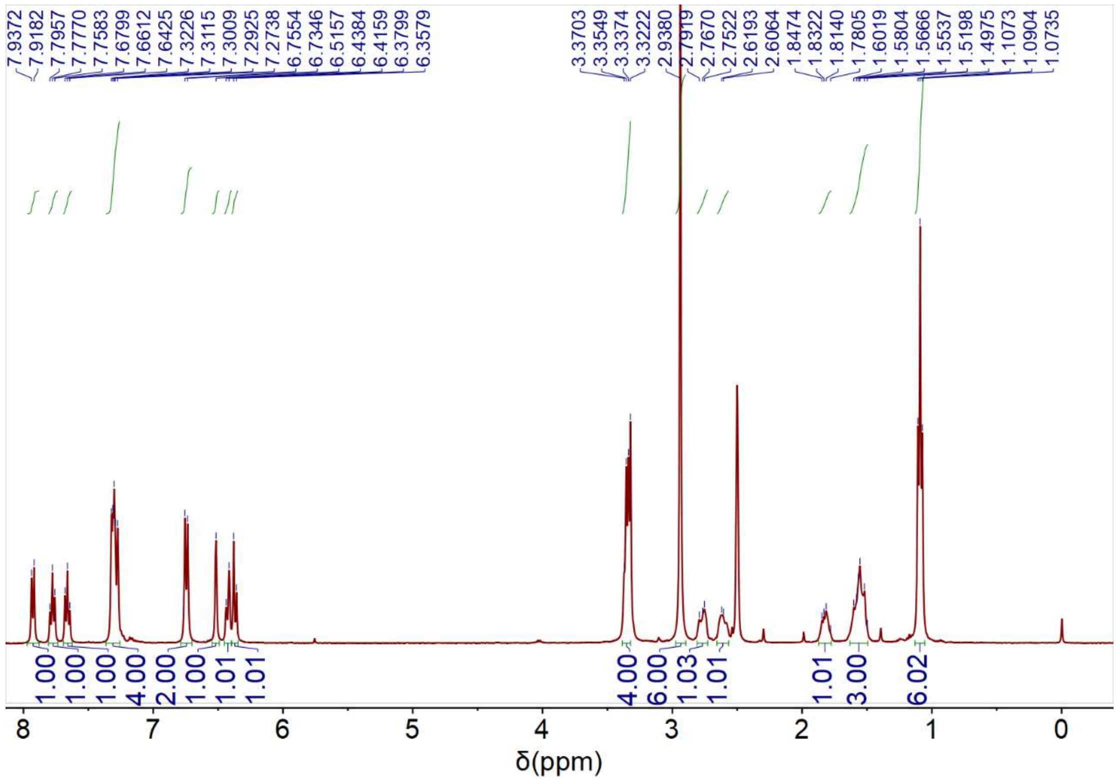

Fig. S22. ^{1}H NMR spectrum (400 MHz, DMSO-d_6) of M15.

Acquisition Parameter

Source Type	ESI	Ion Polarity	Positive	Set Nebulizer	0.6 Bar
Focus	Active	Set Capillary	4500 V	Set Dry Heater	200 °C
Scan Begin	50 m/z	Set End Plate Offset	-500 V	Set Dry Gas	8.0 l/min
Scan End	1200 m/z	Set Collision Cell RF	100.0 Vpp	Set Divert Valve	Waste

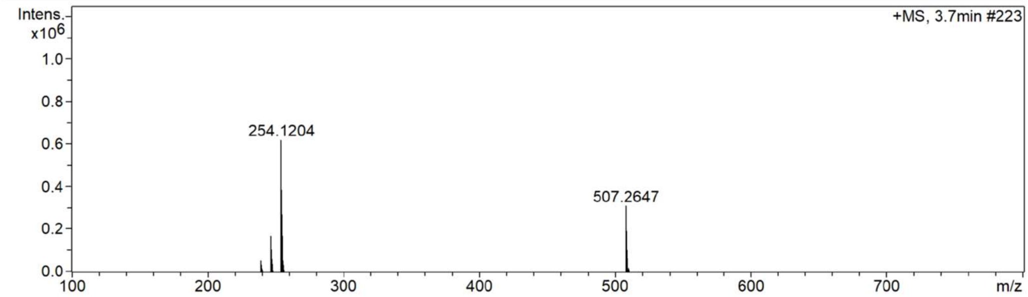

Meas. m/z	#	Formula	m/z	err [ppm]	rdb	N-Rule	e¡¥Conf	mSigma
507.2647								
	1	C 29 H 31 N 8 O	507.2615	-6.1	18.5	ok	even	8.70
	2	C 33 H 35 N 2 O 3	507.2642	-0.9	17.5	ok	even	18.82
	3	C 38 H 35 O	507.2682	7.1	21.5	ok	even	42.88

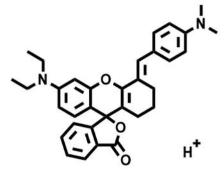

Chemical Formula: $C_{33}H_{35}N_2O_3^+$
Exact Mass: 507.2642
m/z: 507.2642 (100.0%), 508.2676 (35.7%), 509.2709 (3.5%), 509.2709 (2.7%)

Fig. S23. Copy of the mass spectrum report of M15.

Synthesis of M3[4]

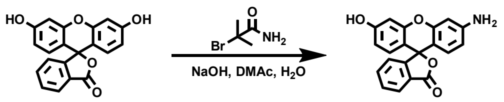

Fluorescein (684 mg, 2.06 mmol) and dimethylacetamide (3 mL) were placed in a flask, and then NaOH (0.247 g, 6.2 mmol) was added, the mixture was stirred at room temperature for 1 h. 2-Bromo-2-methylpropanamide (1.02 g, 6.17 mmol) was added to the solution above, then the mixture was stirred at 30 °C for 10 h. Then NaOH (0.74 g, 18.5 mmol) was added, the mixture was stirred at 55 °C for 1 h. Then water (3 mL) was added and the reaction was heated to reflux for 1 h. Water (6 mL) was added to the mixture and then the reaction was cooled to room temperature. The reaction mixture was poured to water, and the solution was acidified with HCl till the pH of the solution was 3-4, the yellow precipitate was formed then. Extract the solution with EtOAc and DCM, then the organic layer was combined and dried over Na_2SO_4. DCM and MeOH with 0.1% AcOH was used as eluent to separate the product on column chromatography, the ratio of DCM and MeOH was 100/1 at first, and changed to 24/1 gradually and maintained. The product was obtained as orange red solid with a yield of 10 %. ^{1}H NMR (400 MHz, DMSO-d_6) δ 10.06 (s, 1H), 7.96 (d, J = 7.5 Hz, 1H), 7.78 (td, J = 7.5, 1.2 Hz, 1H), 7.69 (td, J = 7.5, 1.0 Hz, 1H), 7.24 (d, J = 7.6 Hz, 1H), 6.64 (d,

J = 2.0 Hz, 1H), 6.52 – 6.47 (m, 2H), 6.40 (d, *J* = 2.0 Hz, 1H), 6.34 (d, *J* = 8.5 Hz, 1H), 6.30 (dd, *J* = 8.6, 2.0 Hz, 1H), 5.65 (s, 2H). LC-HRMS (ESI) calcd. for $C_{20}H_{13}NO_4$ $[M+H]^+$: 332.0917, found: 332.0920.

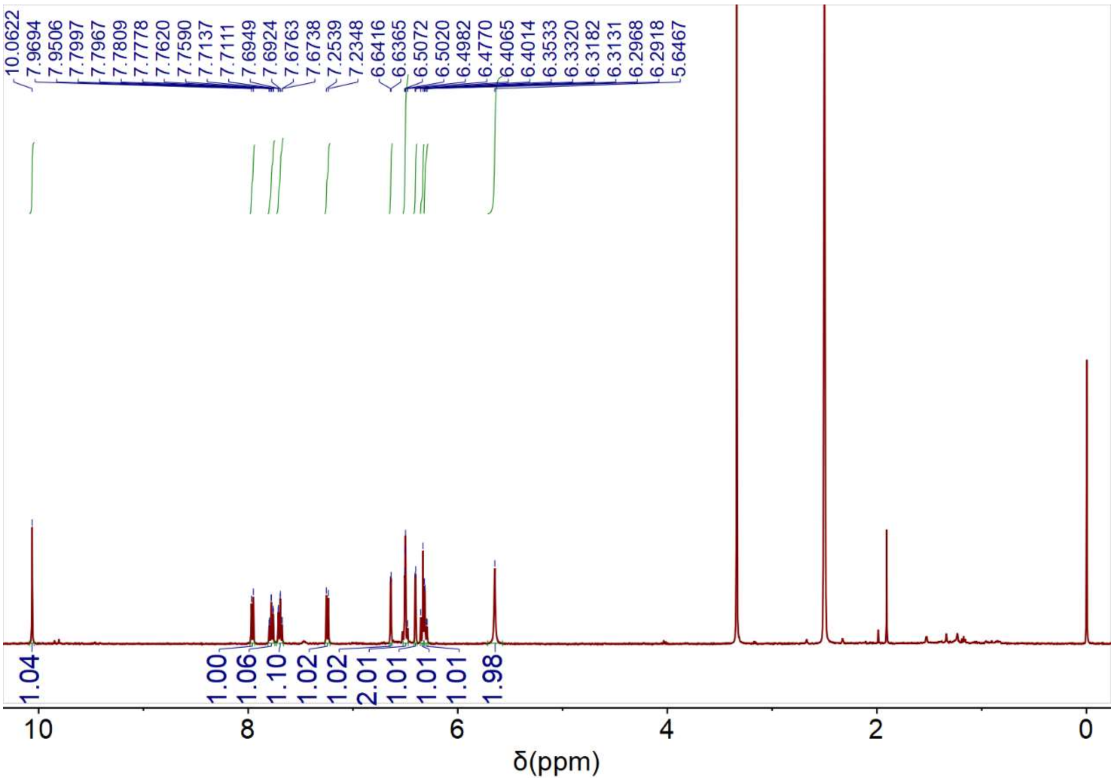

Fig. S24. ^{1}H NMR spectrum (400 MHz, DMSO-d_6) of M3.

Acquisition Parameter

Source Type	ESI	Ion Polarity	Positive	Set Nebulizer	0.4 Bar
Focus	Active	Set Capillary	4500 V	Set Dry Heater	200 °C
Scan Begin	50 m/z	Set End Plate Offset	-500 V	Set Dry Gas	5.0 l/min
Scan End	1200 m/z	Set Collision Cell RF	100.0 Vpp	Set Divert Valve	Waste

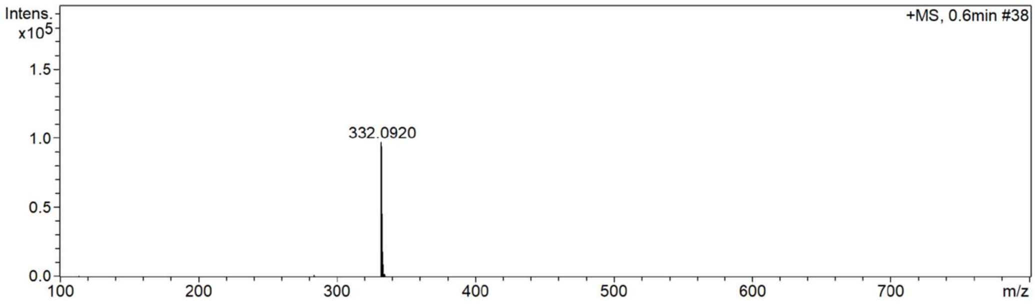

Meas. m/z	#	Formula	m/z	err [ppm]	rdb	N-Rule	e¡¥Conf	mSigma
332.0920								
	1	C 16 H 10 N 7 O 2	332.0890	-9.0	15.5	ok	even	2.99
	2	C 20 H 14 N O 4	332.0917	-0.9	14.5	ok	even	15.09
	3	C 21 H 10 N 5	332.0931	3.1	19.5	ok	even	29.55

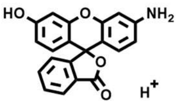

Chemical Formula: $C_{20}H_{14}NO_4^+$
Exact Mass: 332.0917
m/z: 332.0917 (100.0%), 333.0951 (21.6%), 334.0984 (2.2%)

Fig. S25. Copy of the mass spectrum report of M3.

Synthesis of M8

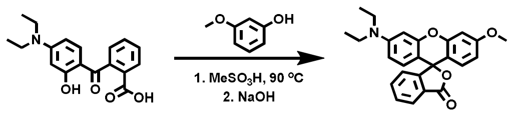

2-(4-(Diethylamino)-2-hydroxybenzoyl)benzoic acid (9.40 g, 30 mmol) was dissolved in 100 mL methyl sulfonic acid and heated to 90 °C, then 3-methoxyphenol (4.10 g, 33 mmol) was added to the solution. The reaction was carried out at 90 °C for 9 h. After cooled to room temperature, the reaction mixture was poured to crushed ice (200 g), NaOH was used to neutralize the acid and dissolve the unreacted reactants in water. The solution was extracted with EtOAc for several times. The organic layer was combined and washed with 1 M NaOH twice and dried over Na_2SO_4. Then column chromatography was performed using PE/EtOAc/N(Et)$_3$ as eluent to obtain the product as light pink solid (yield= 46.5%). ^{1}H NMR (400 MHz, DMSO-d_6) δ 7.99 (d, J = 7.6 Hz, 1H), 7.78 (t, J = 7.4 Hz, 1H), 7.71 (t, J = 7.4 Hz, 1H), 7.26 (d, J = 7.6 Hz, 1H), 6.89 (d, J = 2.3 Hz, 1H), 6.68 (dd, J = 8.8, 2.3 Hz, 1H), 6.63 (d, J = 8.8 Hz, 1H), 6.50 – 6.43 (m, 3H), 3.80 (s, 3H), 3.35 (q, J = 6.7 Hz, 4H), 1.09 (t, J = 6.9 Hz, 6H). LC-HRMS (ESI) calcd. for $C_{25}H_{23}NO_4$ [M+H]$^+$: 402.1700, found: 402.1698.

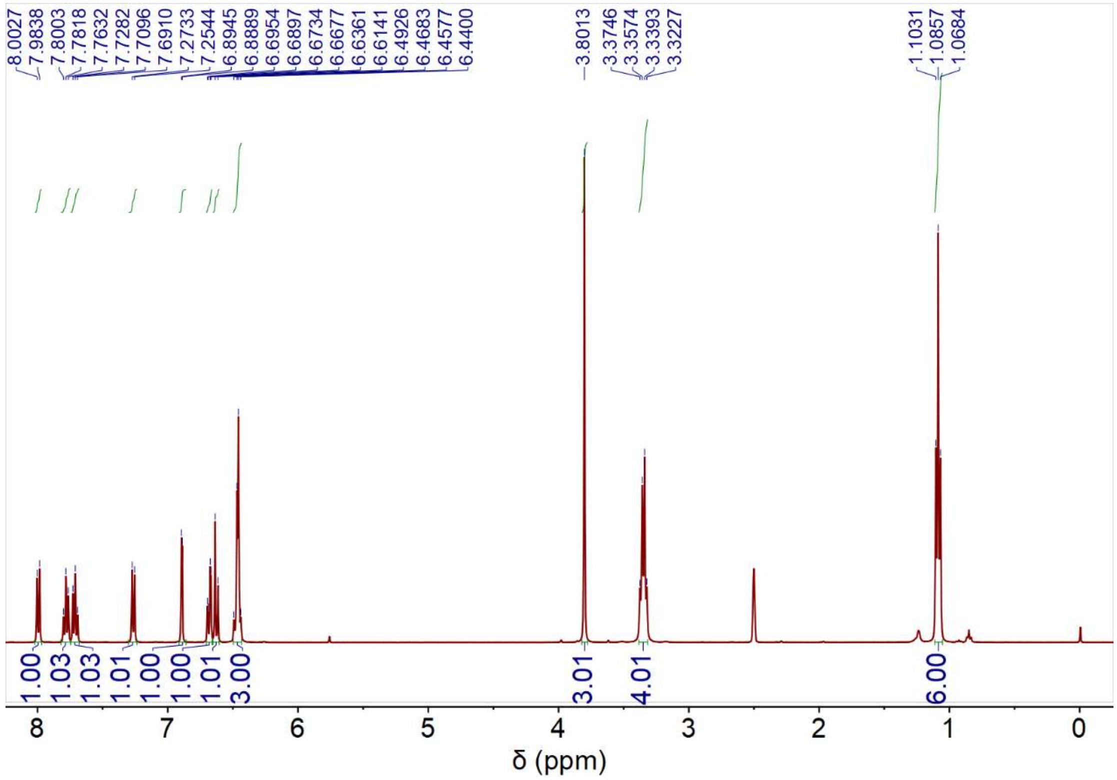

Fig. S26. ^{1}H NMR spectrum (400 MHz, DMSO-d_6) of M8.

Acquisition Parameter

Source Type	ESI	Ion Polarity	Positive	Set Nebulizer	0.6 Bar
Focus	Active	Set Capillary	4500 V	Set Dry Heater	200 °C
Scan Begin	50 m/z	Set End Plate Offset	-500 V	Set Dry Gas	8.0 l/min
Scan End	1200 m/z	Set Collision Cell RF	100.0 Vpp	Set Divert Valve	Waste

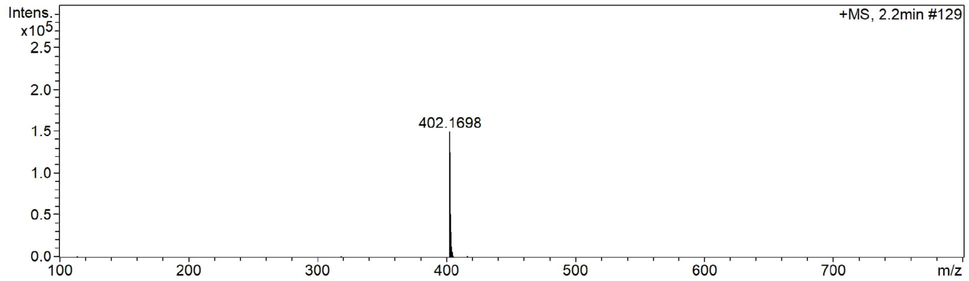

Meas. m/z	#	Formula	m/z	err [ppm]	rdb	N-Rule	ej¥Conf	mSigma
402.1698								
	1	C 21 H 20 N 7 O 2	402.1673	-6.2	15.5	ok	even	6.95
	2	C 20 H 24 N 3 O 6	402.1660	-9.5	10.5	ok	even	11.77
	3	C 25 H 24 N O 4	402.1700	0.5	14.5	ok	even	16.04
	4	C 26 H 20 N 5	402.1713	3.8	19.5	ok	even	30.82

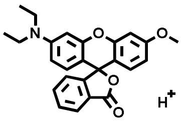

Chemical Formula: $C_{25}H_{24}NO_4^+$
Exact Mass: 402.1700
m/z: 402.1700 (100.0%), 403.1733 (27.0%), 404.1767 (2.7%)

Fig. S27. Copy of the mass spectrum report of M8.

Synthesis of M2[5]

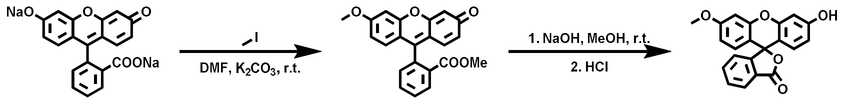

Fluorescein sodium (7.53 g, 20 mmol) and DMF (60 mL) were mixed in a flask, and K_2CO_3 (8.29 g, 60 mmol) was added, then the solution was stirred for 10 min. Iodomethane (8.52 g, 60 mmol) was added to the solution slowly, then the reaction was carried out at room temperature for 12 h. Afterwards, the reaction mixture was poured to crushed ice (200 g), and then stirred for 1 h. The precipitate was collected by filtration under reduced pressure, and washed with water twice. The product was obtained as reddish yellow solid (yield= 85%). The product was used in next step without further purification.

The reactant was dissolved in MeOH (70 mL), then 2 M NaOH (20 mL) was added. The solution was stirred at room temperature for 12 h. The MeOH was removed under reduced pressure, the residue was removed to a beaker, 1 M HCl was used to neutralize the base till a lot of precipitate formed, the precipitate was collected and washed with water twice. Then the product was obtained through column chromatography using PE/EtOAc/AcOH as eluent, the product was yellow solid with yield of 95%. ^{1}H NMR (400 MHz, DMSO-d_6) δ 10.17 (s, 1H), 8.00 (d, J = 7.5 Hz, 1H), 7.79 (t, J = 7.5 Hz, 1H), 7.72 (t, J = 7.4 Hz, 1H), 7.27 (d, J = 7.6 Hz, 1H), 6.94 (d, J = 2.4 Hz, 1H), 6.72

– 6.67 (m, 2H), 6.64 (d, *J* = 8.8 Hz, 1H), 6.57 (s, 2H), 3.81 (s, 3H). LC-HRMS (ESI) calcd. for $C_{21}H_{14}O_5$ $[M+H]^+$: 347.0914, found: 347.0917.

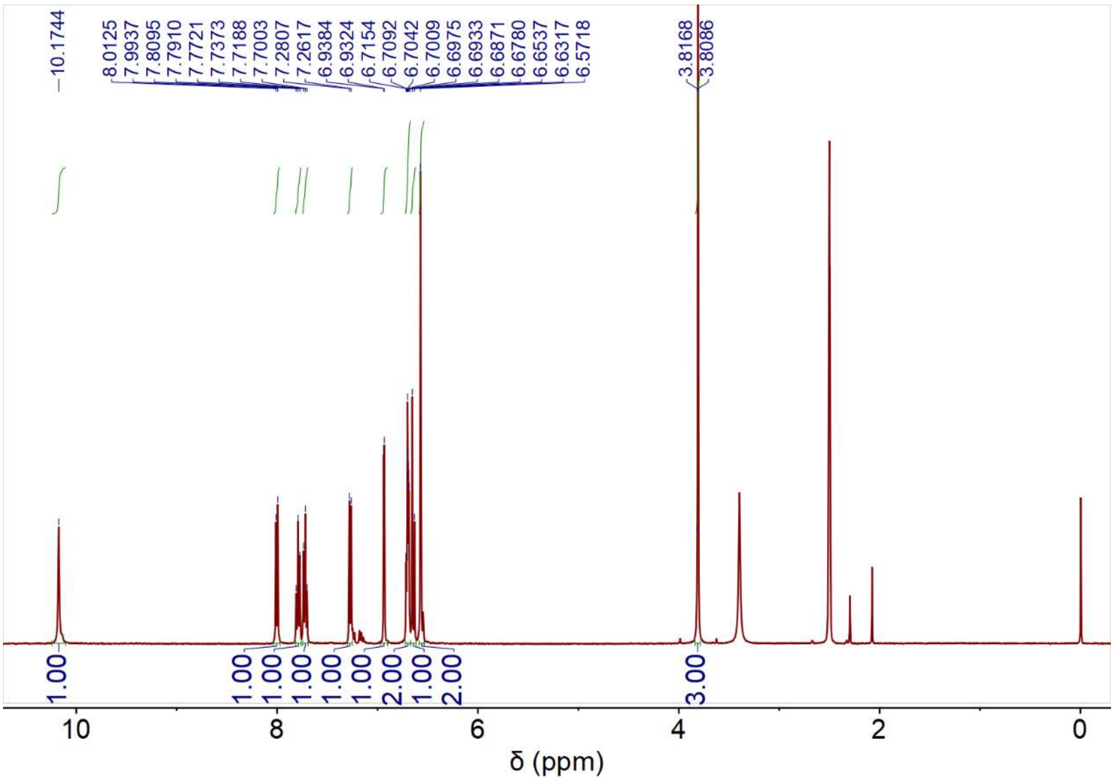

Fig. S28. ^{1}H NMR spectrum (400 MHz, DMSO-d_6) of M2.

Acquisition Parameter

Source Type	ESI	Ion Polarity	Positive	Set Nebulizer	0.6 Bar
Focus	Active	Set Capillary	4500 V	Set Dry Heater	200 °C
Scan Begin	50 m/z	Set End Plate Offset	-500 V	Set Dry Gas	8.0 l/min
Scan End	1200 m/z	Set Collision Cell RF	100.0 Vpp	Set Divert Valve	Waste

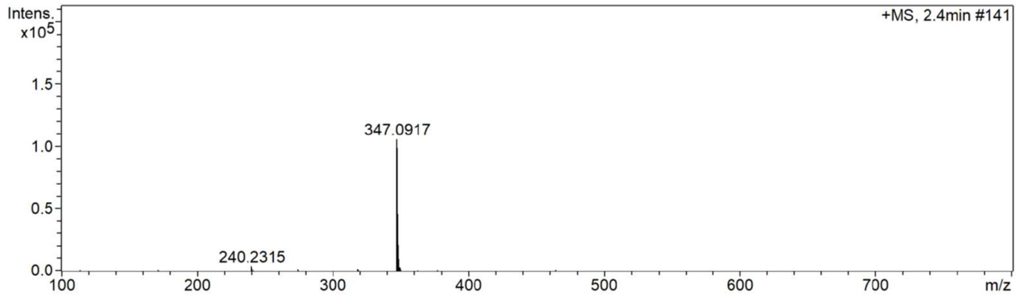

Meas. m/z	#	Formula	m/z	err [ppm]	rdb	N-Rule	e¡¥Conf	mSigma
347.0917								
	1	C 21 H 15 O 5	347.0914	-1.0	14.5	ok	even	6.60
	2	C 17 H 11 N 6 O 3	347.0887	-8.7	15.5	ok	even	6.65
	3	C 22 H 11 N 4 O	347.0927	2.9	19.5	ok	even	21.10

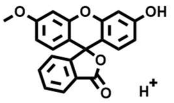

Chemical Formula: $C_{21}H_{15}O_5^+$
Exact Mass: 347.0914
m/z: 347.0914 (100.0%), 348.0948 (22.7%), 349.0981 (2.5%), 349.0956 (1.0%)

Fig. S29. Copy of the mass spectrum report of M2.

Synthesis of M17[6,7]

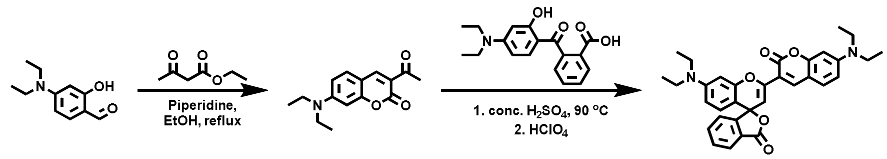

4-(Diethylamino)-2-hydroxybenzaldehyde (3.86 g, 20 mmol), ethyl acetoacetate (3.7 mL, 30 mmol) and piperidine (0.2 mL) were dissolved in ethanol (100 mL), then the reaction was carried out under reflux for 5 h. After cooled to room temperature, the precipitate was formed. The precipitate was collected through filtration, and washed with ethanol twice. The product was obtained as yellow solid with yield of 87.2 %. ^{1}H NMR (400 MHz, DMSO-d_6) δ 8.48 (s, 1H), 7.66 (d, J = 9.0 Hz, 1H), 6.79 (dd, J = 9.0, 2.3 Hz, 1H), 6.57 (d, J = 2.1 Hz, 1H), 3.49 (q, J = 7.0 Hz, 4H), 2.52 (s, 3H), 1.15 (t, J = 7.0 Hz, 6H).

The product above (259 mg, 1 mmol) and 2-(4-diethylamino-2-hydroxybenzoyl)benzoic acid (313 mg, 1 mmol) were added to a flask, then concentrated H_2SO_4 (5 mL) was added, the reaction was carried out at 90 °C for 6 h. After cooled to room temperature, the reaction mixture was poured to crushed ice (10 g), then $HClO_4$ (70 %, 0.5 mL) was added. The solution was decanted out, and the precipitate was washed with water twice. Then saturated Na_2CO_3 solution was used to adjust the pH of the solution to 10, then the solution was extracted with DCM and EtOAc. The organic phase was combined and dried, then the product was separated by column chromatography using PE/EtOAc/N(Et)$_3$ as eluent, the product was obtained as light green solid with yield of 48.5 %. ^{1}H NMR (500 MHz, DMSO-d_6) δ 8.65 (s, 1H), 7.93 (d, J = 7.7 Hz, 1H), 7.78 (td, J = 7.5, 1.0 Hz, 1H), 7.70 – 7.66 (m, 2H), 7.36 (d, J = 7.7 Hz, 1H), 6.82 (dd, J = 9.0, 2.4 Hz, 1H), 6.63 (d, J = 1.5 Hz, 1H), 6.59 (d, J = 2.2 Hz, 1H), 6.51 – 6.46 (m, 2H), 6.36 (s, 1H), 3.48 (q, J = 7.0 Hz, 4H), 3.37 (q, J = 7.0 Hz, 4H), 1.16 – 1.10 (m, 12H). LC-HRMS (ESI) calcd. for $C_{33}H_{32}N_2O_5$ $[M+H]^+$: 537.2384, found: 537.2388.

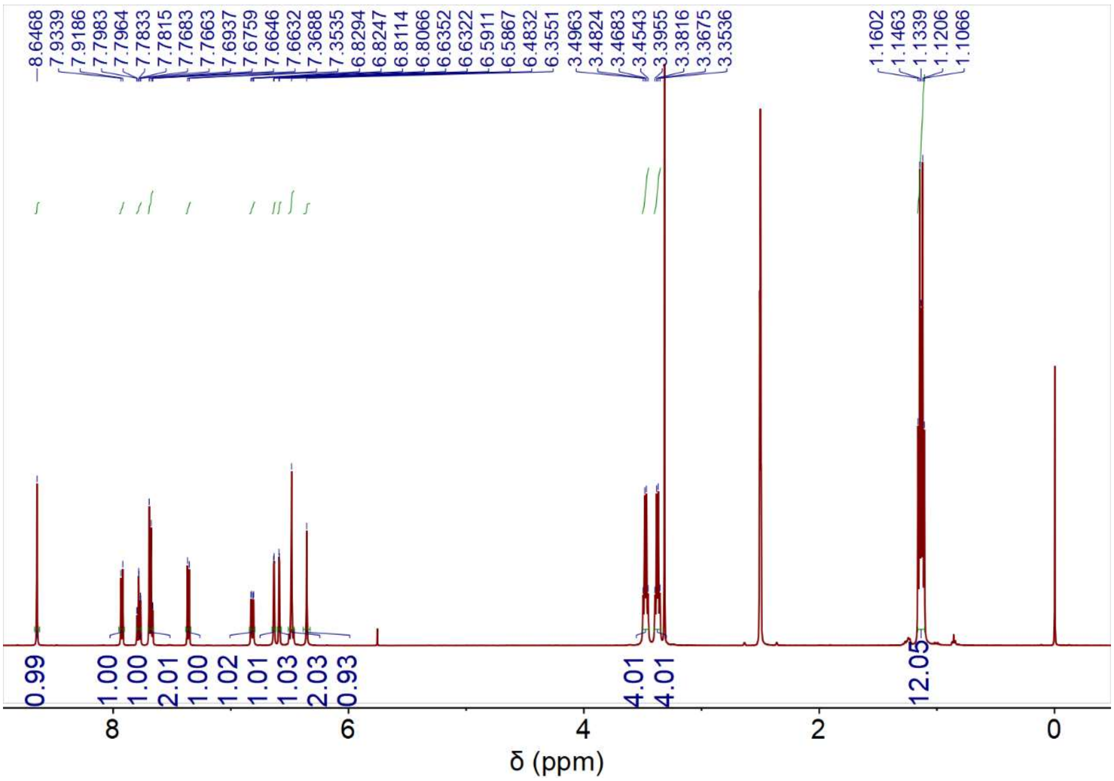

Fig. S30. ^{1}H NMR spectrum (500 MHz, DMSO-d_6) of M17.

Acquisition Parameter

Source Type	ESI	Ion Polarity	Positive	Set Nebulizer	0.4 Bar
Focus	Active	Set Capillary	4500 V	Set Dry Heater	200 °C
Scan Begin	50 m/z	Set End Plate Offset	-500 V	Set Dry Gas	5.0 l/min
Scan End	1200 m/z	Set Collision Cell RF	100.0 Vpp	Set Divert Valve	Waste

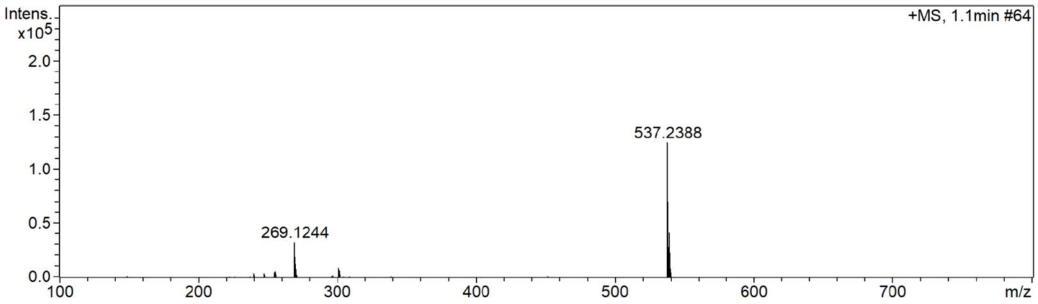

Meas. m/z	#	Formula	m/z	err [ppm]	rdb	N-Rule	e¡¥Conf	mSigma
537.2388								
	1	C 29 H 29 N 8 O 3	537.2357	-5.7	19.5	ok	even	4.69
	2	C 33 H 33 N 2 O 5	537.2384	-0.7	18.5	ok	even	17.58
	3	C 34 H 29 N 6 O	537.2397	1.8	23.5	ok	even	27.99
	4	C 38 H 33 O 3	537.2424	6.8	22.5	ok	even	41.97

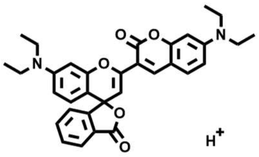

Chemical Formula: $C_{33}H_{33}N_2O_5^+$
Exact Mass: 537.2384
m/z: 537.2384 (100.0%), 538.2418 (35.7%), 539.2451 (6.2%), 539.2426 (1.0%)

Fig. S31. Copy of the mass spectrum report of M17.

Synthesis of M5[8]

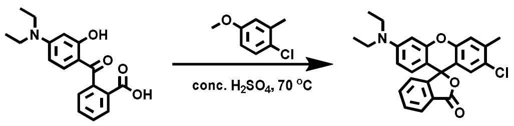

2-(4-(Diethylamino)-2-hydroxybenzoyl)benzoic acid (6.27 g, 20 mmol) and 1-chloro-4-methoxy-2-methylbenzene (3.41 g, 22 mmol) were dissolved in concentrated H_2SO_4 (60 mL), the reaction mixture was stirred at room temperature for 0.5 h. Then the reaction mixture was stirred at 70 °C for 6 h. The solution was poured into crushed ice (200 g), the precipitate was formed, then the liquid was decanted out, the precipitate was washed with water twice. The precipitate was removed to a flask, then NaOH solution (20%, 150 mL) and toluene (200 mL) were added. The mixture was heated to reflux for 2 h. After the mixture cooled to room temperature, the organic layer was collected. The aqueous layer was extracted with toluene twice. The organic phase was combined, and dried over Na_2SO_4. The crude product was obtained after removal of solvent under reduced pressure. Then the product was obtained after recrystallization with toluene as light pink solid (yield= 62.6%). ^{1}H NMR (400 MHz, DMSO-d_6) δ 8.02 (d, J = 7.7 Hz, 1H), 7.81 (t, J = 7.4 Hz, 1H), 7.74 (t, J = 7.5 Hz, 1H), 7.40 (s, 1H), 7.31 (d, J = 7.6 Hz, 1H), 6.70 (s, 1H), 6.47 (s, 3H), 3.36 (q, J = 6.8 Hz, 4H), 2.36 (s, 3H), 1.08 (t, J = 6.9 Hz, 6H). LC-HRMS (ESI) calcd. for $C_{25}H_{22}ClNO_3$ $[M+H]^+$: 420.1361, found: 420.1368.

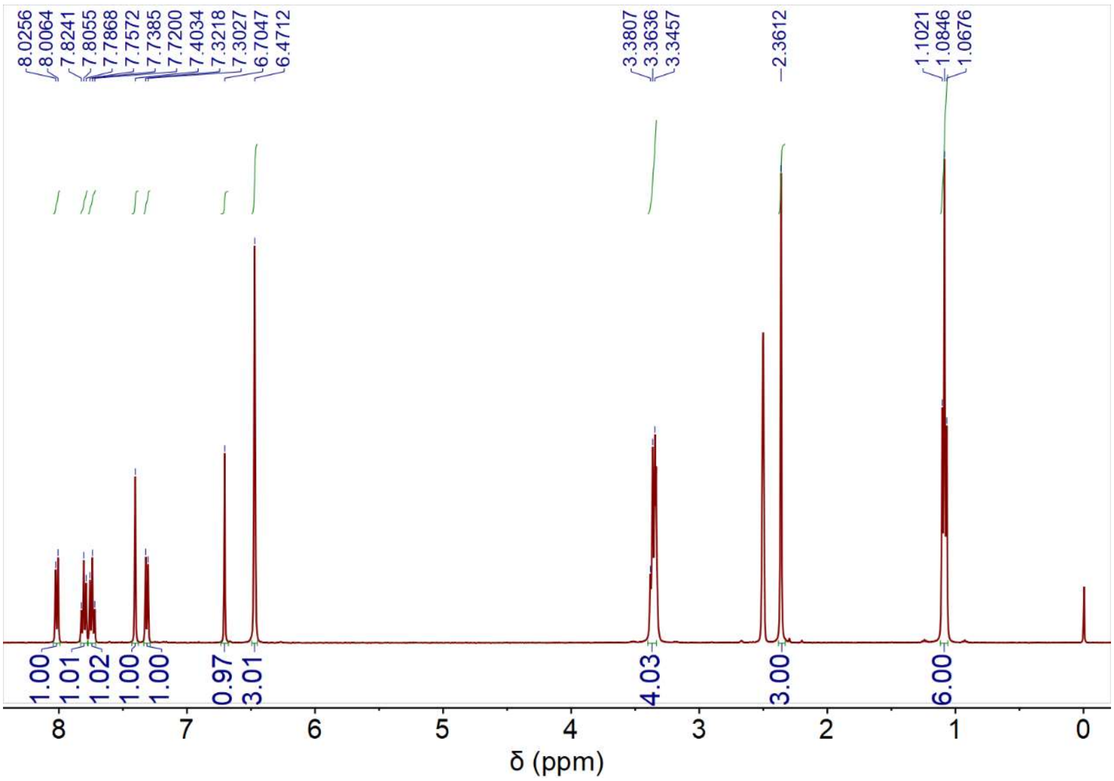

Fig. S32. ^{1}H NMR spectrum (400 MHz, DMSO-d_6) of M5.

Acquisition Parameter

Source Type	ESI	Ion Polarity	Positive	Set Nebulizer	0.8 Bar
Focus	Active	Set Capillary	4500 V	Set Dry Heater	200 °C
Scan Begin	50 m/z	Set End Plate Offset	-500 V	Set Dry Gas	8.0 l/min
Scan End	1200 m/z	Set Collision Cell RF	100.0 Vpp	Set Divert Valve	Waste

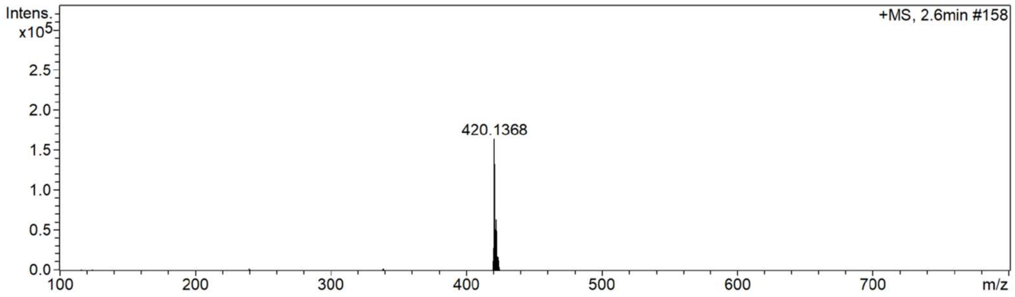

Meas. m/z	#	Formula	m/z	err [ppm]	rdb	N-Rule	e¡¥Conf	mSigma
420.1368								
	1	C 25 H 23 Cl N O 3	420.1361	-1.7	14.5	ok	even	18.63
	2	C 21 H 19 Cl N 7 O	420.1334	-8.1	15.5	ok	even	29.96
	3	C 31 H 18 N O	420.1383	3.5	23.5	ok	even	173.48
	4	C 26 H 18 N 3 O 3	420.1343	-6.1	19.5	ok	even	196.81

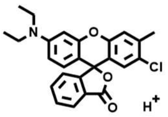

Chemical Formula: $C_{25}H_{23}ClNO_3^+$
Exact Mass: 420.1361
m/z: 420.1361 (100.0%), 422.1331 (32.0%), 421.1395 (27.0%), 423.1365 (8.6%), 422.1428 (3.5%)

Fig. S33. Copy of the mass spectrum report of M5.

Synthesis of M4[8]

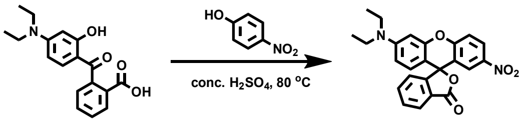

2-(4-(Diethylamino)-2-hydroxybenzoyl)benzoic acid (6.2 g, 20 mmol) and 4-nitrophenol (3.0 g, 22 mmol) were dissolved in concentrated H_2SO_4 (60 mL), the reaction mixture was stirred at room temperature for 0.5 h. Then the reaction mixture was stirred at 80 °C for 20 h. The solution was poured into crushed ice (200 g), then the liquid was decanted out, the precipitate was washed with water twice. The precipitate was removed to a flask, then NaOH solution (20%, 100 mL) and toluene (200 mL) were added. The mixture was heated to reflux for 2 h. After the mixture cooled to room temperature, the organic layer was collected. The aqueous layer was extracted with toluene twice. The organic phase was combined, and dried over Na_2SO_4. The crude product was obtained after removal of solvent under reduced pressure. Then the product was obtained after recrystallization with toluene as yellow solid (yield= 74.0%). ^{1}H NMR (400 MHz, DMSO-d_6) δ 8.33 (dd, J = 9.2, 2.7 Hz, 1H), 8.08 (d, J = 7.5 Hz, 1H), 7.86 (t, J = 7.2 Hz, 1H), 7.79 (t, J = 7.3 Hz, 1H), 7.63 (d, J = 9.2 Hz, 1H), 7.53 (d, J = 2.7 Hz, 1H), 7.42 (d, J = 7.5 Hz, 1H), 6.58 – 6.53 (m, 3H), 3.38 (q, J = 6.9 Hz, 4H), 1.10 (t, J = 6.9 Hz, 6H). LC-HRMS (ESI) calcd. for $C_{24}H_{20}N_2O_5$ [M+H]$^+$: 417.1445, found:

417.1451.

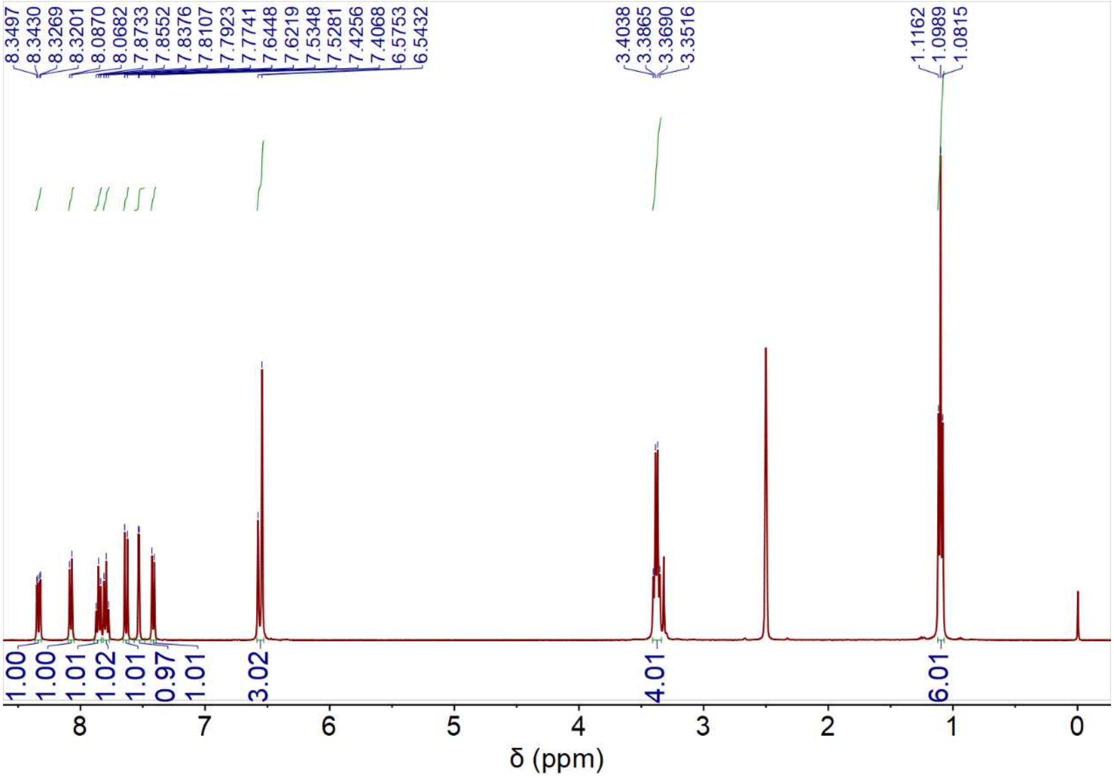

Fig. S34. ^{1}H NMR spectrum (400 MHz, DMSO-d_6) of M4.

Acquisition Parameter

Source Type	ESI	Ion Polarity	Positive	Set Nebulizer	0.8 Bar
Focus	Active	Set Capillary	4500 V	Set Dry Heater	200 °C
Scan Begin	50 m/z	Set End Plate Offset	-500 V	Set Dry Gas	8.0 l/min
Scan End	1200 m/z	Set Collision Cell RF	100.0 Vpp	Set Divert Valve	Waste

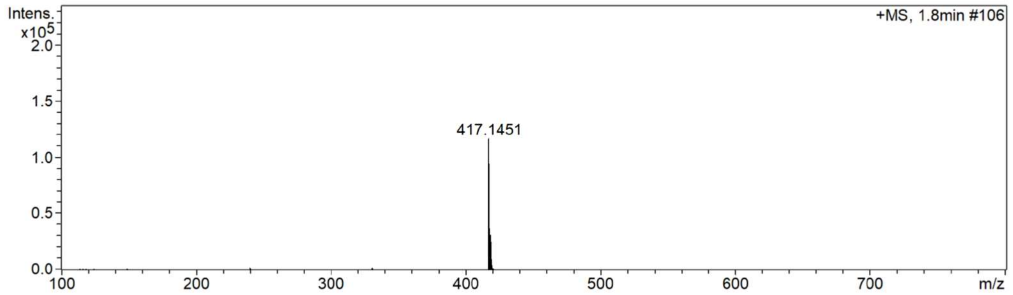

Meas. m/z	#	Formula	m/z	err [ppm]	rdb	N-Rule	e¡¥Conf	mSigma
417.1451								
	1	C 24 H 21 N 2 O 5	417.1445	-1.5	15.5	ok	even	2.31
	2	C 25 H 17 N 6 O	417.1458	1.7	20.5	ok	even	13.00
	3	C 20 H 17 N 8 O 3	417.1418	-7.9	16.5	ok	even	14.56
	4	C 29 H 21 O 3	417.1485	8.2	19.5	ok	even	22.58

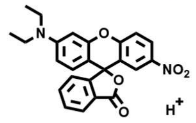

Chemical Formula: $C_{24}H_{21}N_2O_5^+$
Exact Mass: 417.1445
m/z: 417.1445 (100.0%), 418.1479 (26.0%), 419.1512 (2.7%), 419.1487 (1.0%)

Fig. S35. Copy of the mass spectrum report of M4.

Synthesis of M18[3]

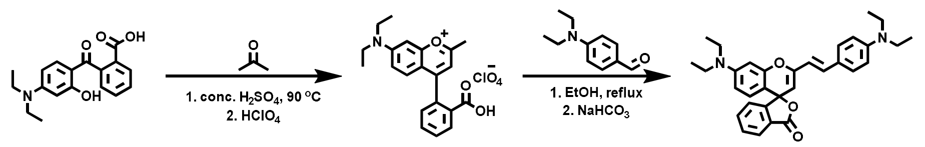

Acetone (3.70 g, 63.7 mmol) was added to concentrated H_2SO_4 (70 mL) dropwise, then the solution was cooled to 0 °C. 2-(4-(Diethylamino)-2-hydroxybenzoyl)benzoic acid (10.03 g, 32 mmol) was added to the solution in portions. Then the solution was heated to 90 °C for 1.5 h and cooled to room temperature slowly. The solution was poured into crushed ice (300 g), and the precipitate was formed after the addition of $HClO_4$ (70%, 7.0 mL). The mixture was let stand for 1 h, then the liquid was decanted off. The precipitate was washed with cold water twice and dried under reduced pressure. The product was obtained as dark red solid (9.59 g). The product was used in next step without further purification.

The reactant (3.36 g, 7.7 mmol) and 4-(diethylamino)benzaldehyde (2.12 g, 12 mmol) were dissolved in ethanol (50 mL), then the solution was fluxed for 6 h. Afterwards, the solvent was removed under reduced pressure. The residue was dissolved in DCM, and washed with saturated $NaHCO_3$ solution twice, then organic phase was dried over Na_2SO_4. The product was obtained after column chromatography using DCM/MeOH/N(Et)$_3$ as eluent. The product was dark green solid and the yield was 28.9%. ^{1}H NMR (400 MHz, DMSO-d_6) δ 7.91 (d, J = 7.6 Hz, 1H), 7.77 (t, J = 7.5 Hz, 1H), 7.66 (t, J = 7.5 Hz, 1H), 7.42 (d, J = 8.6 Hz, 2H), 7.35 (d, J = 7.4 Hz, 1H), 7.26 (d, J = 15.8 Hz, 1H), 6.67 (d, J = 8.7 Hz, 2H), 6.61 (d, J = 16.0 Hz, 1H), 6.55 (s, 1H), 6.52 – 6.41 (m, 2H), 5.32 (s, 1H), 3.37 (q, J = 6.6 Hz, 8H), 1.14 – 1.07 (m, 12H). LC-HRMS (ESI) calcd. for $C_{32}H_{34}N_2O_3$ $[M+H]^+$: 495.2642, found: 495.2641.

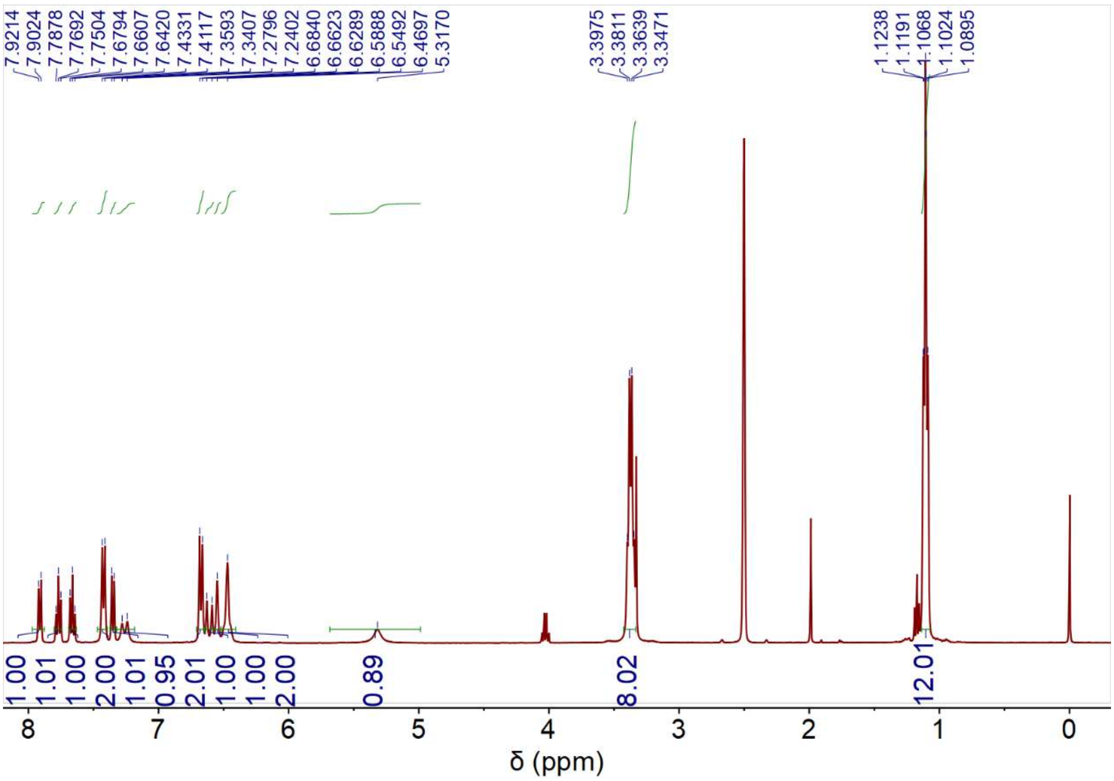

Fig. S36. ^{1}H NMR spectrum (400 MHz, DMSO-d_6) of M18.

Acquisition Parameter

Source Type	ESI	Ion Polarity	Positive	Set Nebulizer	0.8 Bar
Focus	Active	Set Capillary	4500 V	Set Dry Heater	200 °C
Scan Begin	50 m/z	Set End Plate Offset	-500 V	Set Dry Gas	8.0 l/min
Scan End	1200 m/z	Set Collision Cell RF	100.0 Vpp	Set Divert Valve	Waste

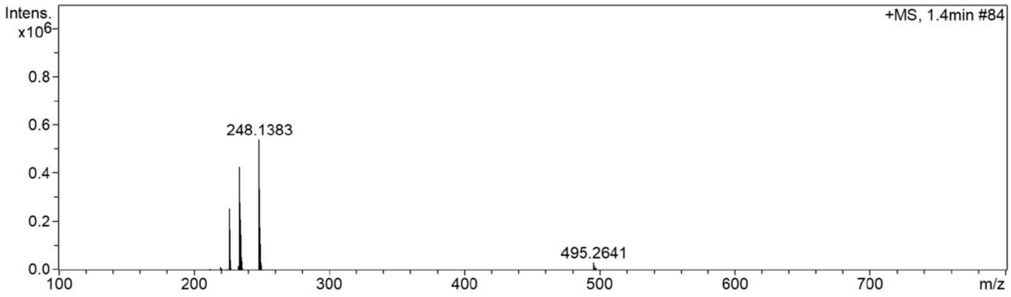

Meas. m/z	#	Formula	m/z	err [ppm]	rdb	N-Rule	e¡¥Conf	mSigma
495.2641								
	1	C 32 H 35 N 2 O 3	495.2642	0.2	16.5	ok	even	10.00
	2	C 28 H 31 N 8 O	495.2615	-5.2	17.5	ok	even	20.95
	3	C 27 H 35 N 4 O 5	495.2602	-7.9	12.5	ok	even	32.91

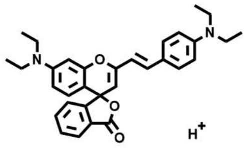

Chemical Formula: $C_{32}H_{35}N_2O_3^+$
Exact Mass: 495.2642
m/z: 495.2642 (100.0%), 496.2676 (34.6%), 497.2709 (3.1%), 497.2709 (2.7%)

Fig. S37. Copy of the mass spectrum report of M18.

Synthesis of M20[9,10]

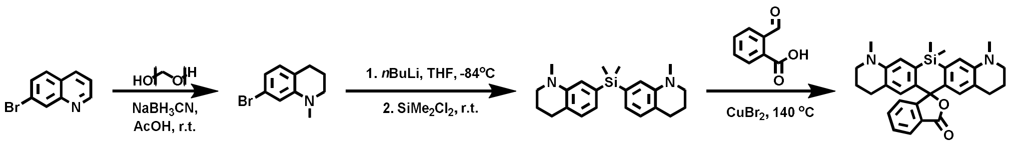

7-Bromoquinoline (416 mg, 2.0 mmol) and paraformaldehyde (600 mg, 20.0 mmol) were dissolved in acetic acid (10 mL), then the solution was cooled to 0 °C. Afterwards, $NaBH_3CN$ (628 mg, 10.0 mmol) was added in portions to the solution above. The solution was stirred at room temperature for 5 h. Then 2 M NaOH was used to neutralize the acid and adjust the pH to 9 under ice-water bath. Ethyl acetate was used to extract the solution twice. The organic phase was combined and dried. The product was separated with column chromatography using PE/EtOAc as eluent. The product was obtained as colorless liquid (yield= 73.9%). ^{1}H NMR (500 MHz, DMSO-d_6) δ 6.80 (d, J = 8.3 Hz, 1H), 6.65 – 6.59 (m, 2H), 3.19 (t, J = 5.7 Hz, 3H), 2.81 (s, 3H), 2.62 (t, J = 6.4 Hz, 2H), 1.85 (p, J = 6.4, 5.7 Hz, 2H).

7-Bromo-1-methyl-1,2,3,4-tetrahydroquinoline (4.11 g, 18.2 mmol) was added into an oven dried flask, then dry tetrahydrofuran (40 mL) was added as solvent. The solvent was cooled to -84 °C, then *n*BuLi (20 mmol in hexane) was added dropwise. The solvent was stirred for 0.5 h at -84 °C. Afterwards, dichlorodimethylsilane (1.17 g, 9.1 mmol) was added dropwise. The solution was allowed to room temperature slowly, and stirred for 2 h. Saturated NH_4Cl was used to quench the reaction. Then the solution was extracted with DCM three times. The organic was combined and dried. The product was obtained through column chromatography using PE/EtOAc as eluent. The product was colorless liquid (yield= 84.7 %). ^{1}H NMR (400 MHz, DMSO-d_6) δ 6.85 (d, J = 7.1 Hz, 2H), 6.67 (s, 2H), 6.63 (d, J = 7.2 Hz, 2H), 3.15 (t, J = 5.5 Hz, 4H), 2.79 (s, 6H), 2.66 (t, J = 6.4 Hz, 4H), 1.86 (p, J = 6.1, 5.6 Hz, 4H), 0.41 (s, 6H).

2-Formylbenzoic acid (705.6 mg, 4.70 mmol) and $CuBr_2$ (21.0 mg, 0.094 mmol) were ground together, and the powder was added to the pressure glass tube. Then dimethylbis(1-methyl-1,2,3,4-tetrahydroquinolin-7-yl)silane (330 mg, 0.94 mmol) was added to the tube. The tube was filled with argon and sealed carefully. Then the reaction was carried out at 140 °C for 0.5 h. After cooled to room temperature, DCM was used to dissolve the solid. The solution was washed with saturated Na_2CO_3 solution and then dried over Na_2SO_4. The product was separated through column chromatography using PE/DCM/EtOAc/N(Et)$_3$ as eluent. The product was white solid with yield of 12 %. ^{1}H NMR (400 MHz, Chloroform-d) δ 7.95 (d, J = 7.3 Hz, 1H), 7.56 (t, J = 7.1 Hz, 1H), 7.48

(t, J = 7.2 Hz, 1H), 7.19 (d, J = 7.4 Hz, 1H), 6.77 (s, 2H), 6.50 (s, 2H), 3.26 – 3.17 (m, 4H), 2.95 (s, 6H), 2.62 – 2.48 (m, 4H), 1.92 – 1.83 (m, 4H), 0.61 (s, 3H), 0.56 (s, 3H). LC-HRMS (ESI) calcd. for $C_{30}H_{32}N_2O_2Si$ $[M+H]^+$: 481.2306, found: 481.2308.

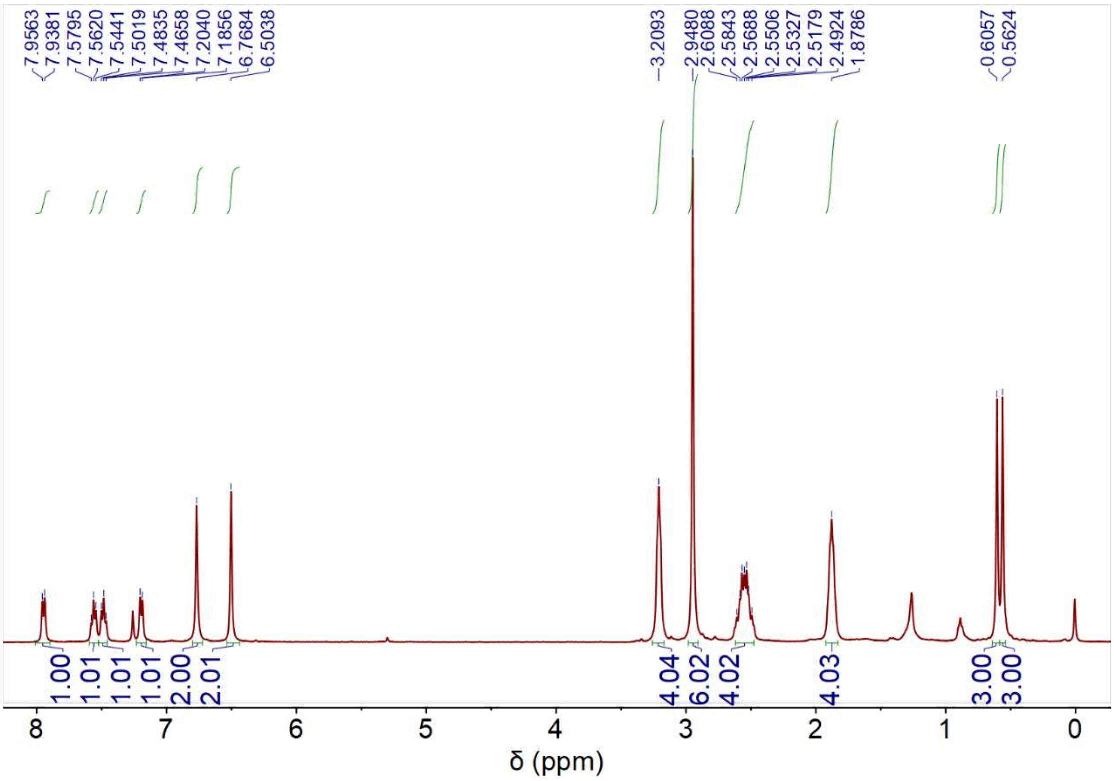

Fig. S38. ^{1}H NMR spectrum (400 MHz, Chloroform-d) of M20.

Acquisition Parameter

Source Type	ESI	Ion Polarity	Positive	Set Nebulizer	0.6 Bar
Focus	Active	Set Capillary	4500 V	Set Dry Heater	200 °C
Scan Begin	50 m/z	Set End Plate Offset	-500 V	Set Dry Gas	8.0 l/min
Scan End	1200 m/z	Set Collision Cell RF	100.0 Vpp	Set Divert Valve	Waste

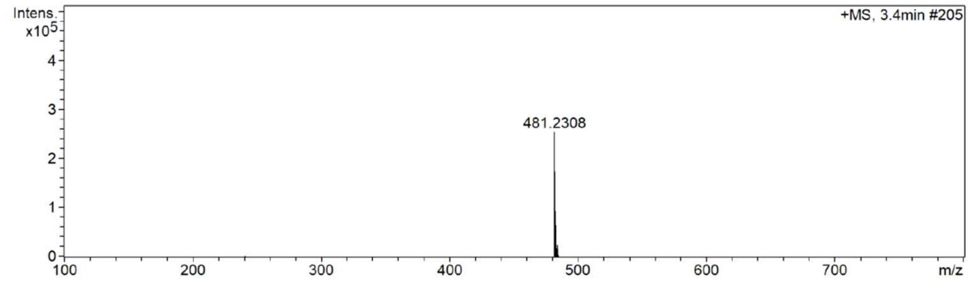

Meas. m/z	#	Formula	m/z	err [ppm]	rdb	N-Rule	e¡¥Conf	mSigma
481.2308								
	1	C 30 H 33 N 2 O 2 Si	481.2306	-0.4	16.5	ok	even	11.98
	2	C 34 H 29 N 2 O	481.2274	-6.9	21.5	ok	even	16.66
	3	C 35 H 33 Si	481.2346	8.0	20.5	ok	even	36.41

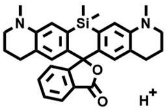

Chemical Formula: $C_{30}H_{33}N_2O_2Si^+$
Exact Mass: 481.2306
m/z: 481.2306 (100.0%), 482.2339 (32.4%), 482.2301 (5.1%), 483.2274 (3.3%), 483.2373 (2.7%), 483.2373 (2.4%), 483.2335 (1.6%), 484.2308 (1.1%)

Fig. S39. Copy of the mass spectrum report of M20.

Synthesis of M22[11]

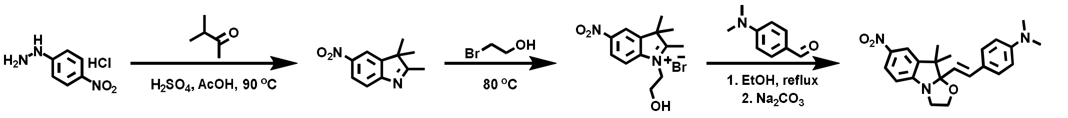

4-Nitrophenylhydrazine hydrochloride (1.9 g, 10 mmol) and 3-methyl-2-butanone (1.29 g, 15 mmol) were dissolved in acetic acid (8 mL), and concentrated H_2SO_4 (1 mL) was added to the solution. The solution was heated at 50 °C for 30 min, then the reaction was carried out at 90 °C for 3 h. After cooled to room temperature, the solution was added saturated Na_2CO_3 under ice-water bath till the pH of solution to 8, the precipitate was formed then. The precipitate was collected through filtration under reduced pressure and washed with cold water, then dried under reduced pressure. The product was obtained as yellowish brown solid (yield= 55.8%).

2,3,3-Trimethyl-5-nitro-3*H*-indole (408.5 mg, 2 mmol), 2-bromoethanol (300 mg, 2.4 mmol) and acetonitrile (8 mL) were added to a flask, then the solution was heated to reflux for 6 h. The reaction mixture was allowed to room temperature, the precipitate was collected through filtration and washed with diethyl ether. The product was obtained as light gray solid (yield= 59.2 %)

The indole salt (0.33 g, 1 mmol) and 4-(dimethylamino)benzaldehyde (0.30 g, 2 mmol) were dissolved in ethanol (2 mL), the air inside the equipment was replaced with nitrogen. The reaction mixture was heated to reflux for 9 h. The reaction was allowed to room temperature, the precipitate was formed. The liquid of the reaction mixture was decanted off. Then the precipitate was washed with EtOAc till the wash solution was colorless. Afterwards, the precipitate was removed to a flask, saturated Na_2CO_3 solution (50 mL) and EtOAc (50 mL) were added to the flask, then the mixture was stirred till the aqueous layer was colorless, where product was dissolved in EtOAc. The EtOAc layer was separated and the aqueous layer was extracted with EtOAc twice. The organic layer was combined and dried over Na_2SO_4. After the removal of solvent, the product was obtained as light yellow solid (yield= 53%). ^{1}H NMR (500 MHz, DMSO-d_6) δ 8.10 (dd, J = 8.7, 2.3 Hz, 1H), 8.02 (d, J = 2.2 Hz, 1H), 7.36 (d, J = 8.7 Hz, 2H), 7.08 (d, J = 8.7 Hz, 1H), 6.71 – 6.64 (m, 3H), 6.03 (d, J = 15.9 Hz, 1H), 3.86 – 3.39 (m, 4H), 2.91 (s, 6H), 1.42 (s, 3H), 1.13 (s, 3H). LC-HRMS (ESI) calcd. for $C_{22}H_{25}N_3O_3$ [M+H]$^+$: 380.1969, found: 380.1972.

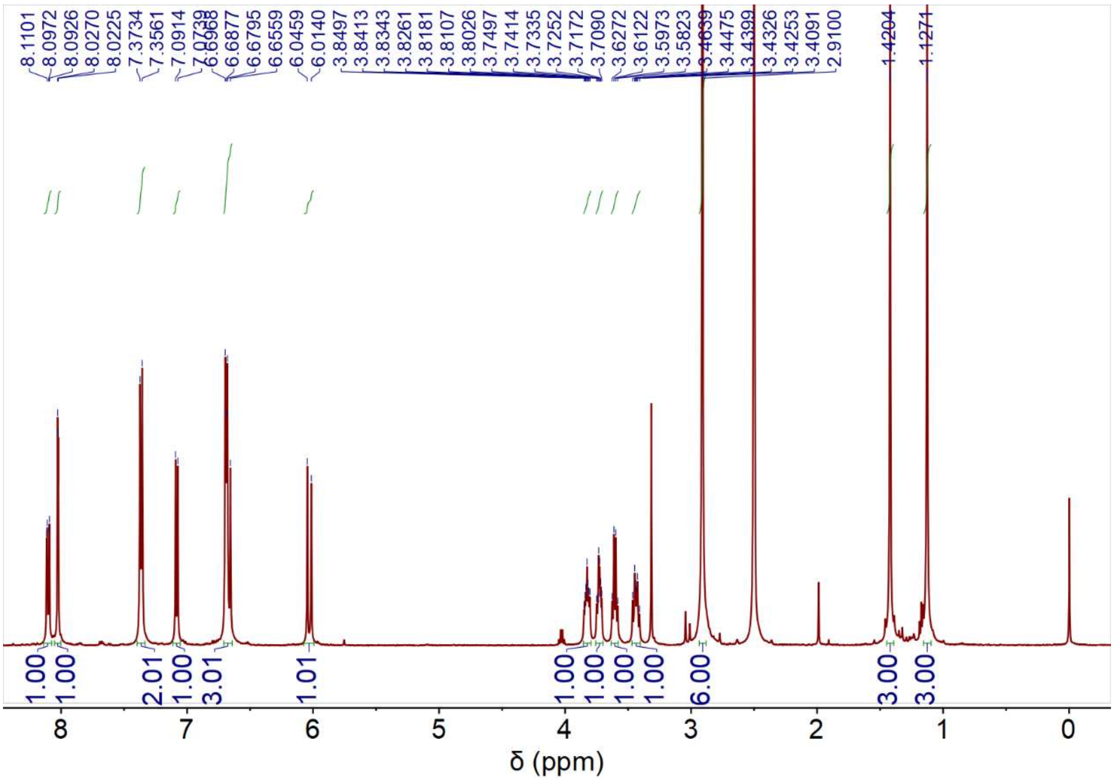

Fig. S40. ^{1}H NMR spectrum (500 MHz, DMSO-d_6) of M22.

Acquisition Parameter

Source Type	ESI	Ion Polarity	Positive	Set Nebulizer	0.6 Bar
Focus	Active	Set Capillary	4500 V	Set Dry Heater	200 °C
Scan Begin	50 m/z	Set End Plate Offset	-500 V	Set Dry Gas	8.0 l/min
Scan End	1200 m/z	Set Collision Cell RF	100.0 Vpp	Set Divert Valve	Waste

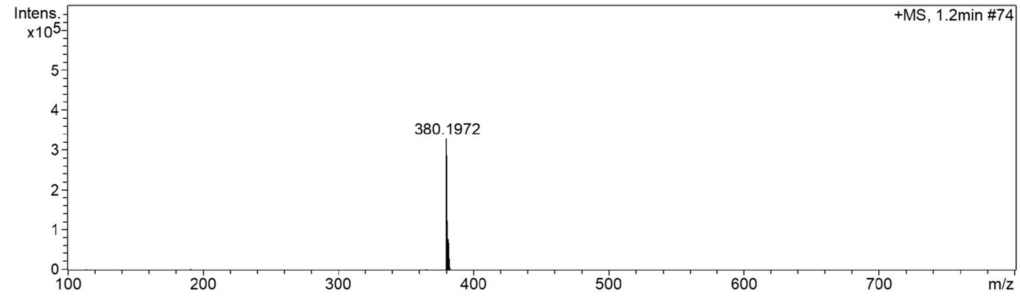

Meas. m/z	#	Formula	m/z	err [ppm]	rdb	N-Rule	e¡¥Conf	mSigma
380.1972								
	1	C 22 H 26 N 3 O 3	380.1969	-0.8	11.5	ok	even	6.94
	2	C 18 H 22 N 9 O	380.1942	-7.9	12.5	ok	even	7.65
	3	C 27 H 26 N O	380.2009	9.8	15.5	ok	even	29.56

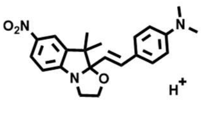

Chemical Formula: $C_{22}H_{26}N_3O_3^+$
Exact Mass: 380.1969
m/z: 380.1969 (100.0%), 381.2002 (23.8%), 382.2036 (2.7%), 381.1939 (1.1%)

Fig. S41. Copy of the mass spectrum report of M22.

Synthesis of M11[1,12]

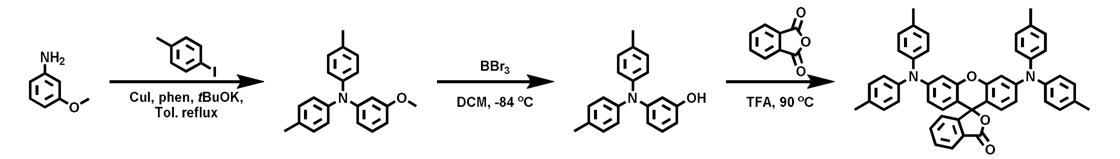

4-Iodotoluene (13.08 g, 60 mmol), 3-methoxyaniline (3.08 g, 25 mmol), CuI (0.48 g, 2.5 mmol), 1,10-phenanthroline (0.45 g, 2.5 mmol), *t*BuOK (8.42 g, 75 mmol) and dry toluene (50 mL) were added to an oven dried flask, the equipment was evacuated and then filled with nitrogen three times. The reaction mixture was heated to reflux for 2 d. The reaction was cooled to room temperature, the solid inside was removed through filtration, the filtrate was collected. Then column chromatography using PE/EtOAc as eluent was used to obtain product as light blond liquid (yield= 66%). ^{1}H NMR (500 MHz, DMSO-d_6) δ 7.15 – 7.09 (m, 5H), 6.90 (d, *J* = 8.4 Hz, 4H), 6.52 (ddd, *J* = 8.2, 2.4, 0.8 Hz, 1H), 6.45 (ddd, *J* = 8.1, 2.1, 0.8 Hz, 1H), 6.38 (t, *J* = 2.3 Hz, 1H), 3.63 (s, 3H), 2.26 (s, 6H).

To an oven dried flask was added 3-methoxy-*N*,*N*-di-*p*-tolylaniline (3.03 g, 10 mmol) and DCM (10 mL), then the solution was cooled to -84 °C. Boron tribromide (5.01 g, 20 mmol) was added to the solution dropwise with stirring under nitrogen. The reaction was stirring at -84 °C for 1 h. Then the reaction was allowed to room temperature slowly, the reaction was stirred at room temperature for 10 h. Under ice-water bath, the solution was added saturated $NaHCO_3$ solution till the pH to 8. DCM was used to extract the product, then the aqueous phase was washed with DCM twice. The product was obtained as light yellow liquid through column chromatography using PE/EtOAc as eluent (yield= 95%). ^{1}H NMR (500 MHz, DMSO-d_6) δ 9.21 (s, 1H), 7.09 (d, *J* = 8.1 Hz, 4H), 7.00 (t, *J* = 8.4 Hz, 1H), 6.89 (d, *J* = 8.4 Hz, 4H), 6.35 – 6.29 (m, 3H), 2.25 (s, 6H).

To a pressure tube was added 3-(di-*p*-tolylamino)phenol (1.75 g, 6 mmol), phthalic anhydride (444.3 mg, 3 mmol) and trifluoroacetic acid (15 mL), the pressure was sealed carefully. The reaction was carried out at 90 °C for 9 h. The reaction was cooled to room temperature, then the solvent was removed under reduced pressure. The residue was dissolved with DCM and washed with saturated $NaHCO_3$ solution. Then column chromatography using PE/EtOAc/$N(Et)_3$ as eluent was used to obtain the product as light green solid (yield= 17%). ^{1}H NMR (400 MHz, Chloroform-*d*) δ 7.97 (d, *J* = 7.6 Hz, 1H), 7.66 (t, *J* = 7.4 Hz, 1H), 7.58 (t, *J* = 7.3 Hz, 1H), 7.25 (d, *J* = 7.6 Hz, 1H), 7.06 (d, *J* = 8.3 Hz, 8H), 7.00 (d, *J* = 8.4 Hz, 8H), 6.65 (d, *J* = 2.2 Hz, 2H), 6.59 (dd, *J* = 8.7, 2.3 Hz, 2H), 6.49 (d, *J* = 8.7 Hz, 2H), 2.29 (s, 12H). ^{13}C NMR (101 MHz, Chloroform-*d*) δ 169.67, 152.97, 152.47, 150.51, 144.36, 134.86, 133.97, 130.22, 129.62, 128.45, 127.42, 125.85, 125.02, 124.24,

116.23, 110.73, 107.27, 84.21, 21.00. Maldi - Tof - MS calcd. for $C_{48}H_{38}N_2O_3$ $[M+H]^+$: 691.296, found: 691.278.

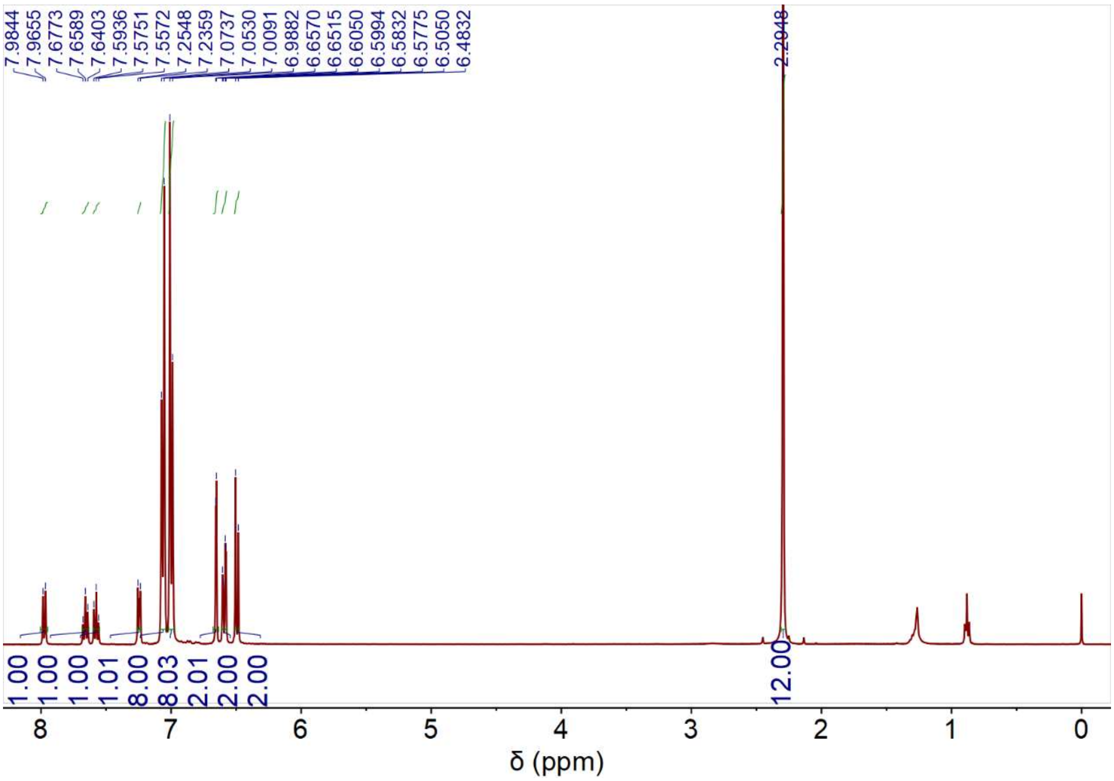

Fig. S42. ^{1}H NMR spectrum (400 MHz, Chloroform-*d*) of M11.

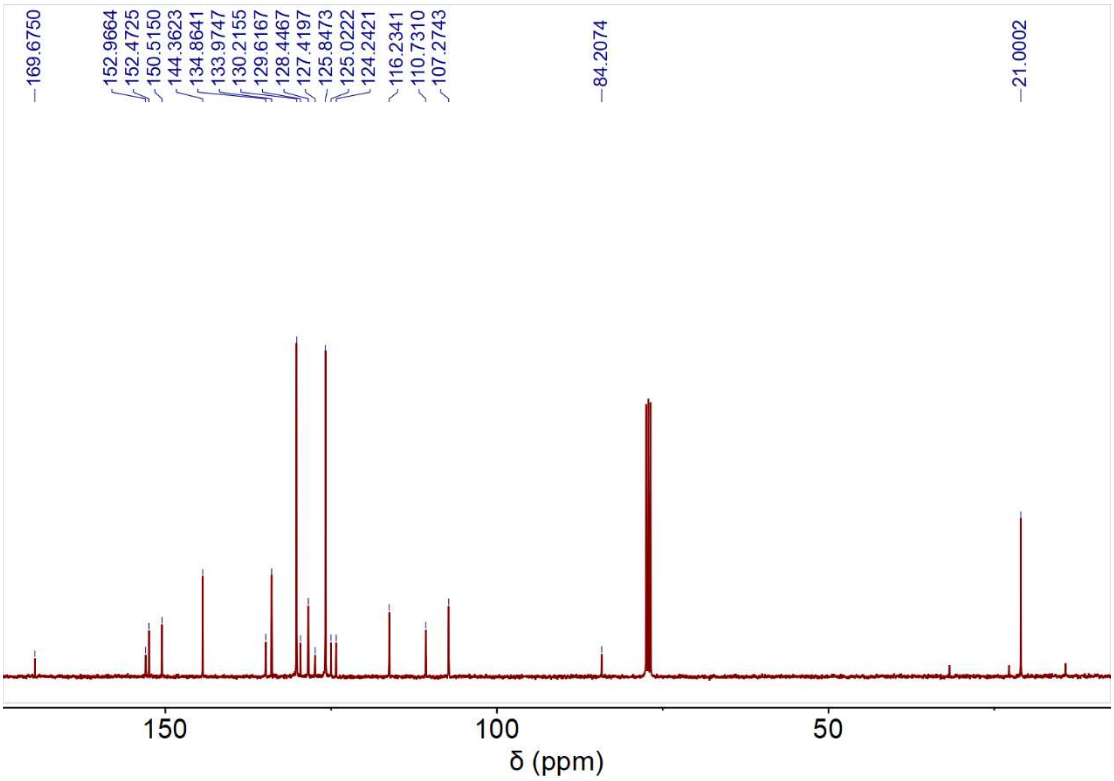

Fig. S43. ^{13}C NMR spectrum (101 MHz, Chloroform-*d*) of M11.

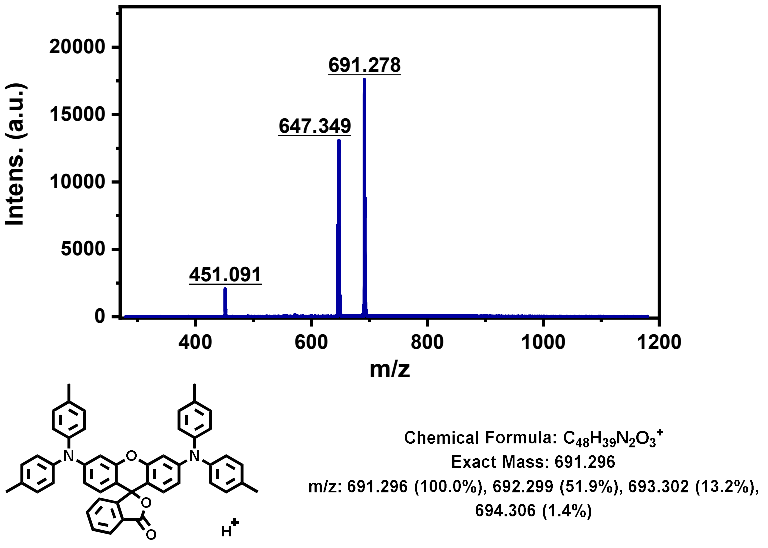

Fig. S44. The mass spectrum report of M11 (Maldi - Tof - MS).

Supplementary reference

[1] Y. Wang, S. Wang, X. Wang, W. Zhang, W. Zheng, Y.-M. Zhang, S. X.-A. Zhang, *Nat. Mater.* **2019**, *18*, 1335.

[2] X. Qu, F. Yuan, Z. He, Y. Mai, J. Gao, X. Li, D. Yang, Y. Cao, X. Li, Z. Yuan, *Dyes Pigments* **2019**, *166*, 72.

[3] K. Zheng, W. Lin, W. Huang, X. Guan, D. Cheng, J.-Y. Wang, *J. Mater. Chem. B* **2015**, *3*, 871.

[4] M. Mizuno, M. Yamano, *Org. Lett.* **2005**, *7*, 3629.

[5] Q. Fu, H. Li, D. Duan, C. Wang, S. Shen, H. Ma, Z. Liu, *Angew. Chem. Int. Ed.* **2020**, *59*, 21546.

[6] J. Liu, Y.-Q. Sun, P. Wang, J. Zhang, W. Guo, *The Analyst* **2013**, *138*, 2654.

[7] S. Ding, Q. Zhang, S. Xue, G. Feng, *The Analyst* **2015**, *140*, 4687.

[8] G. Xi, L. Sheng, J. Du, J. Zhang, M. Li, H. Wang, Y. Ma, S. X.-A. Zhang, *Nat. Commun.* **2018**, *9*, 4819.

[9] Y. Koide, Y. Urano, K. Hanaoka, W. Piao, M. Kusakabe, N. Saito, T. Terai, T. Okabe, T. Nagano, *J. Am. Chem. Soc.* **2012**, *134*, 5029.

[10] Y. Huo, J. Miao, L. Han, Y. Li, Z. Li, Y. Shi, W. Guo, *Chem. Sci.* **2017**, *8*, 6857.

[11] L. Sheng, M. Li, S. Zhu, H. Li, G. Xi, Y.-G. Li, Y. Wang, Q. Li, S. Liang, K. Zhong, S. X.-A. Zhang, *Nat. Commun.* **2014**, *5*, 3044.

[12] A. G. Jadhav, S. Kothavale, N. Sekar, *Dyes Pigments* **2017**, *138*, 56.
